# Recommendations for uniform definitions used in newborn screening for severe combined immunodeficiency

**DOI:** 10.1016/j.jaci.2021.08.026

**Published:** 2021-09-16

**Authors:** Maartje Blom, Rolf H. Zetterstroöm, Asbjørg Stray-Pedersen, Kimberly Gilmour, Andrew R. Gennery, Jennifer M. Puck, Mirjam van der Burg

**Affiliations:** a Department of Pediatrics, Laboratory for Pediatric Immunology, Leiden University Medical Center; b Centre for Inherited Metabolic Diseases, Karolinska University Hospital, Karolinska Institutet, Stockholm; c Department of Molecular Medicine and Surgery, Karolinska Institutet, Stockholm; d Norwegian National Unit for Newborn Screening, London; e Department of Pediatrics, Division of Pediatric and Adolescent Medicine, Oslo University Hospital, London; f University College London Great Ormond Street Institute of Child Health, London; g Great Ormond Street Hospital for Children National Health Service Foundation Trust, London; h National Institute for Health Research–Great Ormond Street Hospital Biomedical Research Center, London; i Children’s Bone Marrow Transplant Unit, Great North Children’s Hospital, Newcastle University, Newcastle upon Tyne; j Translational and Clinical Research Institute, Newcastle University, Newcastle upon Tyne; k Division of Allergy, Immunology, and Blood and Marrow Transplantation, Department of Pediatrics, University of California, San Francisco School of Medicine, San Francisco Benioff Children’s Hospital San Francisco.; l University of California, San Francisco Benioff Children’s Hospital San Francisco.

**Keywords:** Newborn screening, neonatal screening, severe combined immunodeficiency terminology, case definitions, T-cell receptor excisions circles

## Abstract

**Background:**

Public health newborn screening (NBS) programs continuously evolve, taking advantage of international shared learning. NBS for severe combined immunodeficiency (SCID) has recently been introduced in many countries. However, comparison of screening outcomes has been hampered by use of disparate terminology and imprecise or variable case definitions for non-SCID conditions with T-cell lymphopenia.

**Objectives:**

This study sought to determine whether standardized screening terminology could overcome a Babylonian confusion and whether improved case definitions would promote international exchange of knowledge.

**Methods:**

A systematic literature review highlighted the diverse terminology in SCID NBS programs internationally. While, as expected, individual screening strategies and tests were tailored to each program, we found uniform terminology to be lacking in definitions of disease targets, sensitivity, and specificity required for comparisons across programs.

**Results:**

The study’s recommendations reflect current evidence from literature and existing guidelines coupled with opinion of experts in public health screening and immunology. Terminologies were aligned. The distinction between *actionable* and *nonactionable* T-cell lymphopenia among non-SCID cases was clarified, the former being infants with T-cell lymphopenia who could benefit from interventions such as protection from infections, antibiotic prophylaxis, and live-attenuated vaccine avoidance.

**Conclusions:**

By bringing together the previously unconnected public health screening community and clinical immunology community, these SCID NBS deliberations bridged the gaps in language and perspective between these disciplines. This study proposes that international specialists in each disorder for which NBS is performed join forces to hone their definitions and recommend uniform registration of outcomes of NBS. Standardization of terminology will promote international exchange of knowledge and optimize each phase of NBS and follow-up care, advancing health outcomes for children worldwide.

In the past decade, newborn screening (NBS) for severe combined immunodeficiency (SCID), the most profound inborn error of immunity (IEI), has been introduced in many screening programs worldwide.^1,2^ Prompt clinical intervention with hematopoietic stem cell transplantation (HSCT) or gene therapy is required to prevent morbidity and early mortality for these patients.^3,4^ SCID is the first immune disorder to be accepted for population-based screening, and implementation has provided important clinical benefits for affected infants as well as lessons for public health programs, immunologists, and pediatricians.

NBS for SCID is based on quantification of the molecular biomarker T-cell receptor excision circle (TREC), a byproduct of the normal recombination of the T-cell receptor genes as thymocytes differentiate into mature T cells.^5^ TRECs are quantitated by PCR in DNA isolated from infant dried blood spots (DBSs). Infants with SCID lack T cells, and consequently, the absence of TRECs in their DBSs identifies SCID with remarkable sensitivity.^6^ However, other non-SCID conditions associated with T-cell lymphopenia in the neonatal period are also identified as having fewer TRECs than normal, leading to reduced specificity that must be addressed by each individual SCID NBS program.^7,8^ In NBS for SCID, case definitions for actionable T-cell lymphopenia, nonactionable T-cell lymphopenia, and secondary findings have not previously been clearly defined.

Public health programs have the responsibility to continuously optimize NBS for their stakeholders. International shared learning will expedite effective implementation of SCID screening for all infants. However, when sharing experiences, a challenging hurdle has arisen. Comparison of screening algorithms, cutoff values, and referral policies, as well as uniform registration of cases with abnormal screening results, have to date been hampered by differing terminology between NBS programs. Simply said, “it’s a mess,” and there is a need for standardization of screening terminology to avoid a Babylonian confusion.

Our group, representing specialists with direct experience in screening, clinical immunology, and pediatrics has used SCID to illustrate the divergence of screening terms used in NBS programs for SCID worldwide. With the aid of a systematic literature search and existing guidelines, we considered the range of terminologies for reporting NBS test results, screening strategies, case definitions, and clinical outcomes. Most importantly, we suggest uniform definitions for SCID screening test outcomes and diagnostic follow-through to be used in scientific publications and registries. These recommendations are designed to aid all screening programs, uniting the SCID screening community with the clinical immunology community, while suggesting a critical reevaluation of case definitions used for other screened disorders as well as SCID.

## METHODS

### Systematic review

**FIG 1.**
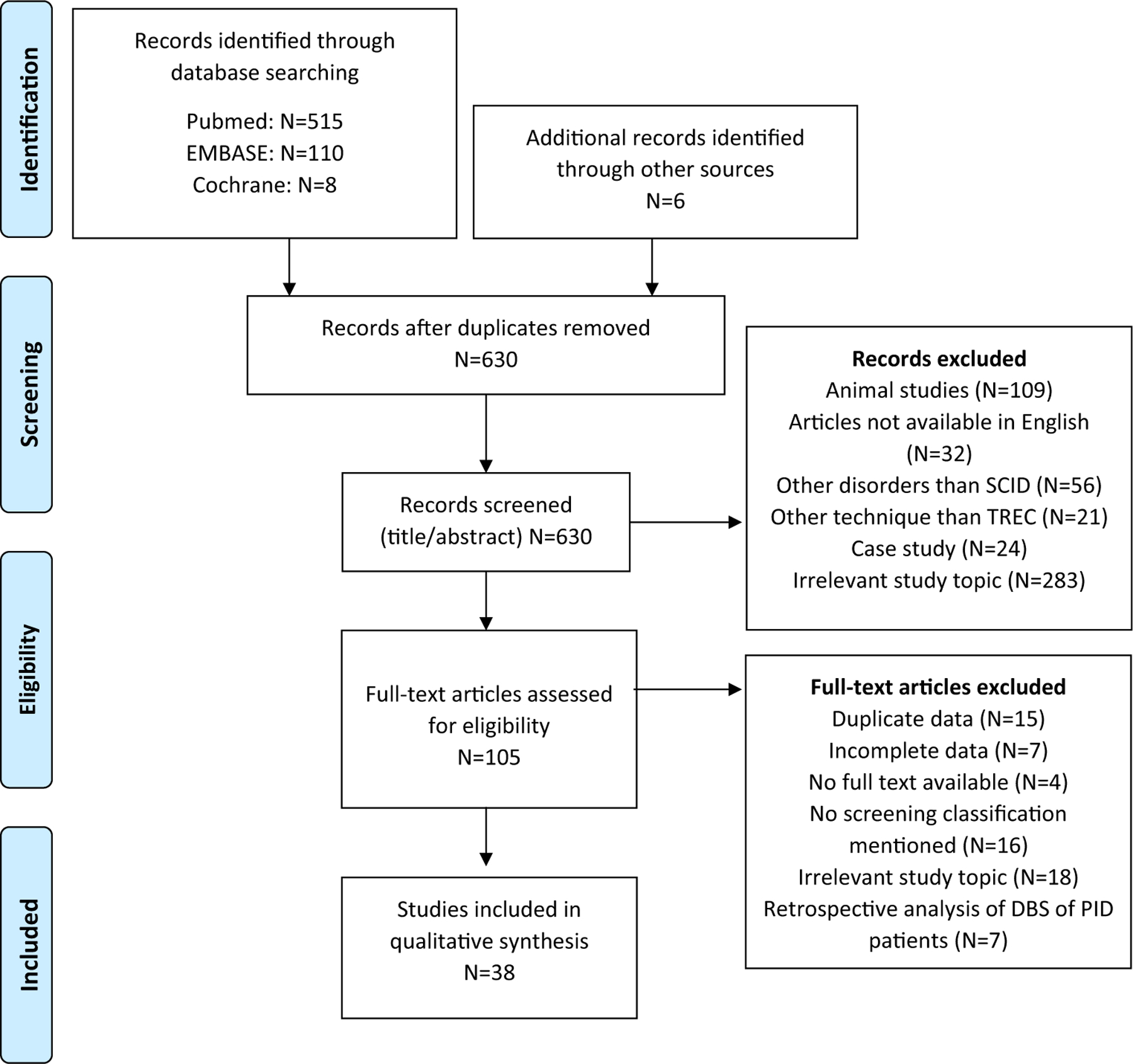
Flow diagram used for article selection in the systematic review of definitions used in NBS for SCID. Search performed on February 16, 2021. *PID*, Primary immune deficiency.

A systematic review was conducted on NBS for SCID and case definitions used in pilot studies and population-based screening. An electronic search was performed on MEDLINE (PubMed), EMBASE (excluding MEDLINE), Cochrane library, and Scopus databases. The search strategy is shown in the Online Repository (available at www.jacionline.org). The study selection flow diagram is shown in Fig 1. The eligibility criteria, study selection, data extraction, and quality assessment are specified in the Online Repository.

### Guidelines and panel

Existing guidelines of the European Society for Immunodeficiencies (ESID),^9^ the Association of Public Health Laboratories (APHL),^10^ the Clinical and Laboratory Standards Institute (CLSI),^11^ Primary Immune Deficiency Treatment Consortium experience (PIDTC),^12^ Clinical Immunology Society, Immune Deficiency Foundation (IDF),^13^ and International Union of Immunological Societies (IUIS)^14,15^ were evaluated and considered when formulating recommendations. Meetings were held with leading experts in the field of NBS for SCID, IEI, immunological diagnostics, genetics, and stem cell transplantation. The panel, consisting of 7 members from 5 different countries, came together after a virtual meeting on NBS for SCID organized by the International Society for Neonatal Screening and the United Kingdom Newborn Screening Laboratory Network. Each member brought his/her own expertise and experience in NBS for SCID, and together the group formulated consensus-based recommendations reflecting all currently available evidence.

## RESULTS

### NBS programs use different definitions in literature

Our search resulted in 630 unique records. By checking the reference lists of selected articles, we included 6 additional articles. After screening abstracts and titles, 38 articles were included in the qualitive analysis (Fig 1). Four overview articles,^16–19^ 11 population-based studies,^20–30^ 20 pilot studies,^31–50^ and 3 studies including both pilot and population data^51–53^ were included. The number of screened newborns ranged from 141 in Korea^37^ to 3,252,156 in California,^22^ with varying referral and retest rates between screening programs. Study characteristics are further specified in Table E1 (see this article’s Online Repository at www.jacionline.org).

### Definitions of screening results used in studies on NBS for SCID

**FIG 2.**
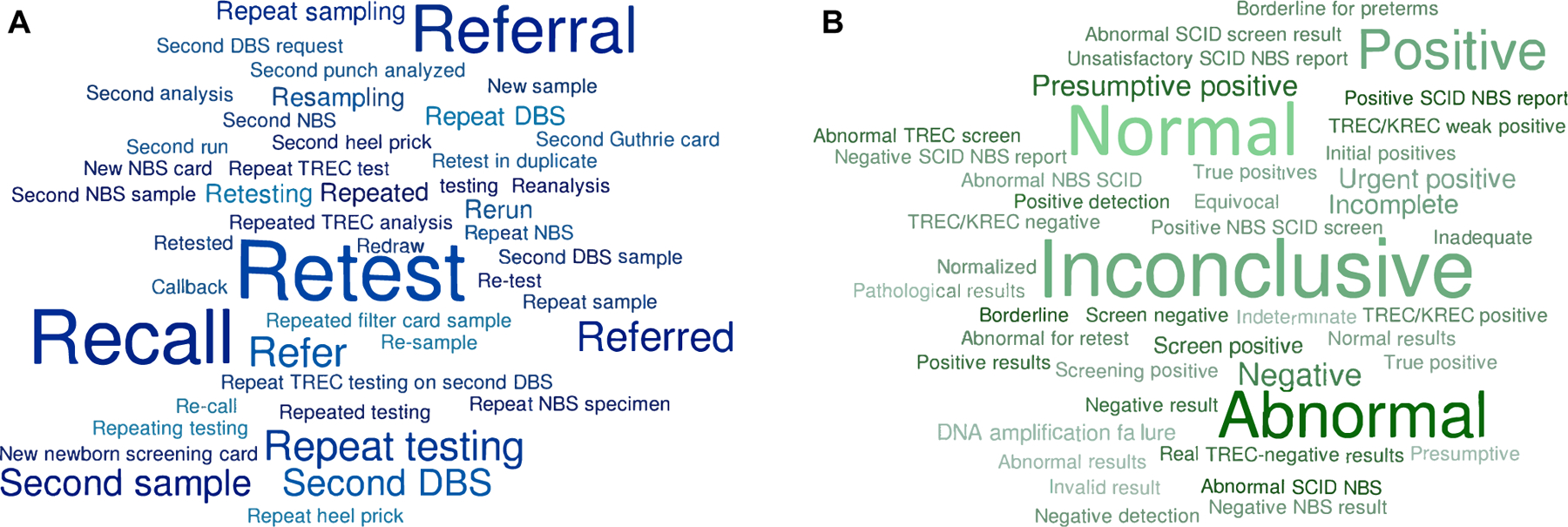
**A**, Different terminology used for screening results in studies on NBS for SCID. Word cloud based on Table E2. **B**, Different terminology used for variables in screening algorithms studies on NBS for SCID. Word cloud based on Table E3.

Definitions predominantly used to describe NBS test results were *negative* or *normal* (TRECs above cutoff) versus *positive* or *abnormal* (TRECs below cutoff) (Fig 2, A). Some programs distinguished between *positive* and *urgent positiv*e test results, with the lowest TREC levels requiring more rapid follow-up actions.^27,31^ One study used the opposite terminology defining TREC *positive* as present TRECs (*Cp* value <37.0) and TREC *negative* as low/absent TRECs (*Cp* value >39.0).^37^ Users of the EnLite TREC-assay (PerkinElmer, Waltham, Mass) often included *presumptive positive* to specify that TRECs were below cutoff after repeated analysis on the same NBS card in duplicate.^16,45,48,52^ *Inconclusive* was the predominant terminology used for failure of internal control amplification, but *indeterminate,*^16^ *incomplete*,^22,27,31^ and *unsatisfactory*^50^ were also described (Fig. 2, A; see Table E2 in this article’s Online Repository at www.jacionline.org).

### Definition of variables in the screening algorithm used in studies on NBS for SCID

There is a range of terms used to describe certain actions in screening algorithms employed at public health screening laboratories. *Retesting* was most commonly used to indicate repeated TREC analysis; most NBS programs perform this analysis on the same NBS card either reusing the original DNA extract or using DNA from a new punch from the same card, while other programs use the term *retest* when requesting a new NBS card from the infant (Fig 2, B; see Table E3 in this article’s Online Repository at www.jacionline.org). Other terms used for *retesting* are *repeat(ed) testing*, *reanalysis*, *duplicate/second analysis*, *rerun*, *second punch analysi*s, and *second run*. Requesting a second NBS card was usually more diversely described by terms such as *second (NBS/DBS) sampling, second Guthrie card, new sample/NBS card, resampling, redraw, second heel prick, second DBS request, repeat NBS/DBS (specimen), repeat sampling,* and so on. To indicate that a newborn with low TREC levels was evaluated by a pediatrician or immunologist with follow-up diagnostics, *referral* was primarily used (Fig 2, B). In contrast, some programs included the term *recall* or *call back,* which could mean an infant recalled for a new DBS sample by a nurse or pediatrician, as well as an infant sent to receive a clinical evaluation, flow cytometric diagnostics, and genetic testing.^41,42^

### Classification of (case) definitions and outcomes after follow-up used in studies on NBS for SCID

Classification of diagnoses or outcomes after an abnormal SCID screening result differed greatly among NBS programs (see Table E4 in this article’s Online Repository at www.jacionline.org). Some programs used their own criteria to define SCID, while others used criteria from existing guidelines such as those published by the PIDTC.^12^ In some, but not all programs, SCID was subclassified into typical, leaky/atypical, and Omenn syndrome. Non-SCID T-cell lymphopenia was generally divided into (1) syndromes that include variable T-cell impairment (or non-SCID T-cell lymphopenia due to syndromes and/or patients who are syndromic); (2) secondary T-cell lymphopenia (or transient T-cell lymphopenia due to a nonimmunologic neonatal condition); and (3) idiopathic T-cell lymphopenia (in some case referred to as *variant SCID*). Premature birth alone was mentioned as a separate outcome category in 15 of 38 studies, but otherwise was included with secondary T-cell lymphopenia. *False-positive* referrals were mentioned in 12 studies, but exact descriptions of the term varied. Finally, some publications listed the status of newborns (eg, flow cytometry pending or lost to follow-up) or all diagnoses without classification, while 5 pilot studies were unable to classify newborns with low TRECs because of anonymized inclusion and no clinical follow-up.

### Definitions of premature infants used in studies on NBS for SCID

The majority of the included studies defined prematurity as a gestational age <37 weeks. Some NBS programs discriminated among moderate, very, and extremely preterm^31^ or included low birth weight (≤2500 g) as an additional parameter.32,45 In other reports, prematurity was mentioned, but not further specified, or not reported at all. Many programs have tried to limit their number of referrals by including adjustments in their screening algorithm for preterm infants with low TREC levels. Countries are requesting second NBS cards when preterm newborns reach a certain gestational age, monitoring preterm infants with serial NBS specimens, or using a lower TREC cutoff value for premature infants (see Table E5 in this article’s Online Repository at www.jacionline.org).

### Guidelines use different definitions

Different guidelines are available to classify NBS SCID outcomes or to help clinicians in diagnosing IEI based on clinical, biological, and genetic features. In addition to published NBS studies, the new uniform definitions for SCID NBS must take immunologic diagnostic criteria into account to ensure that terminology and classifications apply seamlessly for all phases of the screening program from initial DBS testing through diagnosis and outcomes after follow-up.

ESID has developed working definitions for clinical diagnosis of IEI^9^ that can help clinicians with a clinically probable diagnosis of an individual who is symptomatic being evaluated prior to genetic testing. The criteria include invasive or opportunistic infections or other symptoms, a positive family history, manifestations of disease early in life, and exclusion of HIV; there are also T-cell–specific laboratory results. ESID provides suggestions for alternative diagnosis if the criteria are not completely fulfilled.^9^

The APHL has provided case definition tables for all disorders included in NBS programs, including SCID.^10^ The SCID definitions were created by a panel of experts between 2011 and 2013 and updated in 2018. A distinction was made between the primary target of NBS (typical SCID, leaky SCID, and Omenn syndrome) and secondary targets (syndromes with variable immune defects with some cases having significantly low T-cell numbers, secondary T-cell lymphopenia, and idiopathic T-cell lymphopenia). The primary target diagnoses are classified as *definitive, probable, possible*, or *uncertain* based on CD3 T cells/μL, proliferation to PHA, maternal engraftment, molecular testing, and clinical presentation. For non-SCID T-lymphopenic conditions, maternal engraftment would be absent, T cells might be largely naïve (bearing the surface marker CD45RA or equivalent) and PHA proliferation would usually be normal.^10^

The CLSI provided a guideline for NBS for SCID by measurement of TRECs in 2013, including a chapter on terminology and definitions (NBS06-A).^11^ Distinctions were made among (1) typical SCID, (2) leaky SCID and Omenn syndrome, (3) variant SCID, (4) syndromes with primary T-cell lymphopenia, (5) secondary T-cell lymphopenia not due to prematurity alone, and (6) preterm infants. Diagnoses in these categories were further explained in the appendix of the CLSI document. CLSI also provided definitions for other screening parameters such as false positives/negatives, screen-positive/-negative results, and retests.^11^ A new version of the CLSI guideline is currently being developed.

In 2014, the PIDTC developed a uniform set of criteria for diagnosing SCID and related disorders by an expert group who have seen substantial numbers of SCID cases over many years.^12^ Patients with SCID (n 5 285) were retrospectively assigned to 1 of 3 strata: (1) typical SCID; (2) leaky SCID, Omenn SCID, and reticular dysgenesis; and (3) SCID with non-HSCT treatments. Using strict eligibility criteria,^12^ 86% of patients with SCID or SCID-related conditions could be assigned to one of the established strata. Lack of critical laboratory information led to difficulties in dealing with the remaining 14% of the patients. The experts acknowledged that the criteria might evolve over time and highlighted the increasing role of genotyping in establishing diagnosis, particularly in the setting of NBS.

The Clinical Immunology Society refers to the diagnostic and clinical care guidelines for primary immunodeficiencies from the IDF^13^ and the classification of IUIS.^14^ IDF is a national patient organization that developed these guidelines in partnership with expert immunologists to enhance earlier diagnosis. The IDF distinguishes SCID with reticular dysgenesis, SCID with low T- and B-cell numbers, SCID with low or normal B-cell numbers and other combined immunodeficiencies. In addition, DiGeorge syndrome, ataxia telangiectasia and Wiskott-Aldrich syndrome are also listed under cellular or combined immunodeficiencies.

The IUIS expert committee has published and updated biannually a genotypic and phenotypic classification of all IEIs.^14,15^ This classification is organized into tables, each of which attempts to group IEIs sharing a given pathogenesis and immunologic features. Clinical and laboratory results are used for the diagnostic algorithm and phenotypical classification. SCID and nonsevere combined immunodeficiencies affecting both cellular and humoral immunity already include >50 different disorders caused by mutations in 58 genes. T-cell lymphopenia in SCID is defined by CD3^1^ T cells <300/μL.^14,15^ The IUIS gene lists have grown and become more complex as the discovery of novel IEI disorders has been occurring at an impressive rate. In addition, the clinical spectrum has become broader for many conditions as more patients are observed.^54^

## DISCUSSION

We have aimed to underline the gaps in language and perspective between the NBS community and the field of clinical (diagnostic) immunology. Immunologists have already developed international nomenclature to describe cell phenotypes, enabling easy cross-border communication. A similar language is required for outcomes of NBS SCID to enable comparison of NBS programs. International shared learning between public health programs and immunologists will expedite effective implementation of SCID screening for all infants. There is need to bring these disciplines together by creating shared case definitions to exchange information via uniform registration of screening outcomes in scientific publications and registries to optimize and improve NBS programs worldwide.

### Constraints of individual programs: Harmonization of screening strategies is not required, but uniform registration of screening outcomes is

We acknowledge that there are constraints of individual programs and certain terms have been incorporated in NBS for many years. NBS programs use a variety of test methods, cutoff values, and screening algorithms to balance a high sensitivity, detecting all patients with SCID, while preventing high referral rates in their particular populations. Some programs have included the request of a second NBS card in their screening algorithm, while others have included second-tier tests such as next-generation sequencing.^51^ In addition, other test methods such as tandem mass spectrometry for adenosine deaminase deficiency or purine nucleoside phosphorylase deficiency have been proposed.^55,56^ There is no need to harmonize individual screening strategies; however, to avoid confusion, we recommend uniform designations for screening outcomes independent of how they are generated. NBS programs can use their own definitions in practice, but they are encouraged to conform to uniform terminology when publishing program outcomes internationally.

### Considerations in defining screening terminology

**FIG 3.**
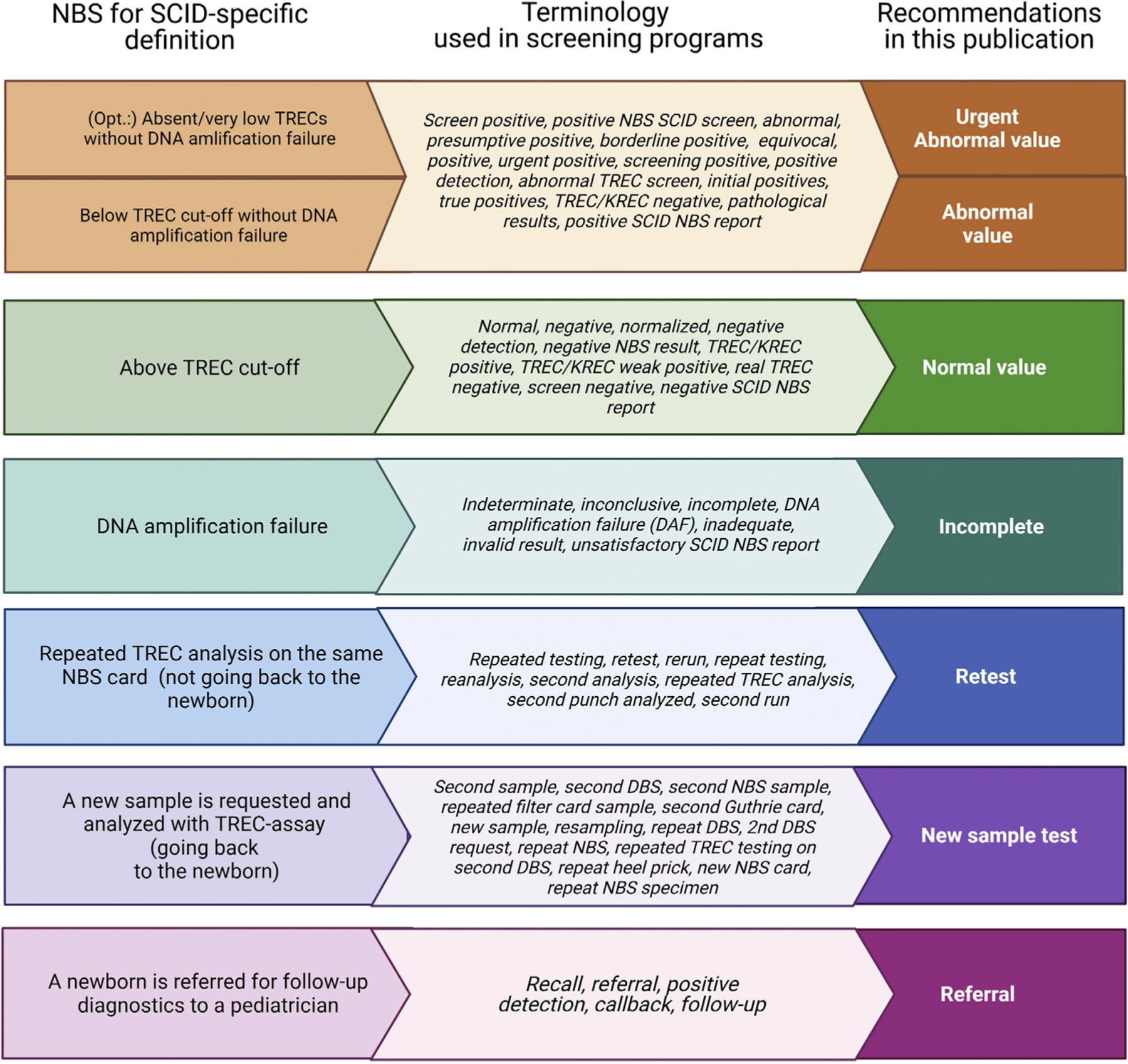
Recommendations on definitions of screening terminology. *KREC*, Kappa-deleting recombination excision circle.

The systematic literature review highlighted the diversity of terminology used in NBS programs. Clear recommendations without ambiguity are required for clinicians, public health specialists, and other NBS stakeholders, such as policy makers and parents. *Positive* and *negative* are commonly used terms in NBS, but definitions vary between programs. “TREC positive” could imply the presence of TRECs, but the term *positive* is also broadly used for a screen with TRECs below cutoff. In addition, families can interpret a positive test result as “positive” or good news. *Abnormal* and *normal* are nonspecific terms that can have negative connotations. Labeling an infant as abnormal causes parental anxiety, while the term normal excludes the fact that newborns can have serious disorders not screened for. The terms *within normal range* or *outside normal range* might be preferred, but ranges are not applicable to SCID NBS because only TRECs below a certain cutoff value are important. We therefore recommend the terms *abnormal value* and *normal value* to describe TREC screening results (Fig 3). *Incomplete* is recommended if further action is required due to DNA amplification failure.

For screening algorithm outcomes, we agree with the term *retest,* which is commonly used in literature. However, it should be specified that retesting is TREC analysis of the same NBS card (not going back to the newborn for a new card). If TREC analysis is repeated on a new NBS card, we feel that the term *new sample test* is best. The term *second NBS card/sample* is not completely correct as some programs are requesting a routine second NBS card for other disorders, such as congenital hypothyroidism, and this new sample to resolve SCID screening could be the third NBS card. It is important to highlight when a new sample is taken from the newborn as repeated sampling is not without anxiety and emotional insecurity for parents and additional distress for the newborn. Finally, we prefer the term *referral* (meaning sending for specialist evaluation) over *recall*, as recall is differently used across programs (Fig 3).

### Considerations in defining diagnostic outcomes after an abnormal value screening test

In addition to unique screening strategies, screening programs for SCID also differ in diagnostic approaches and follow-up of newborns with low TREC levels. Existing guidelines describing diagnostic criteria for SCID and other immunodeficiencies are of great aid to clinicians in facilitating diagnosis of these conditions worldwide. We therefore recommend to define SCID according to the widely used PIDTC guidelines, which also allow subcategorization into leaky SCID and Omenn syndrome.^12^ Even though diagnostic guidelines help immunologists with a prompt and consistent approach to a definitive diagnosis, the translation to the NBS community, which should also include definitions of non-SCID T-cell lymphopenic conditions, is lacking. Thus we recommend to subdivide non-SCID T-cell lymphopenia into 3 categories: (1) syndromes that can be associated with T-cell impairment, (2) reversible conditions with T-cell impairment that resolves on treatment of the underlying cause, and idiopathic T-cell lymphopenia. The term *variant SCID*, originally considered analogous to variant forms of inborn errors of metabolism, should not be used as it does not describe any specific group of patients who are immunodeficient recognized by immunologists; while the term has been applied in the screening phase of SCID NBS programs, it has no counterpart in the diagnostic setting of immunology specialty care.

**FIG 4.**
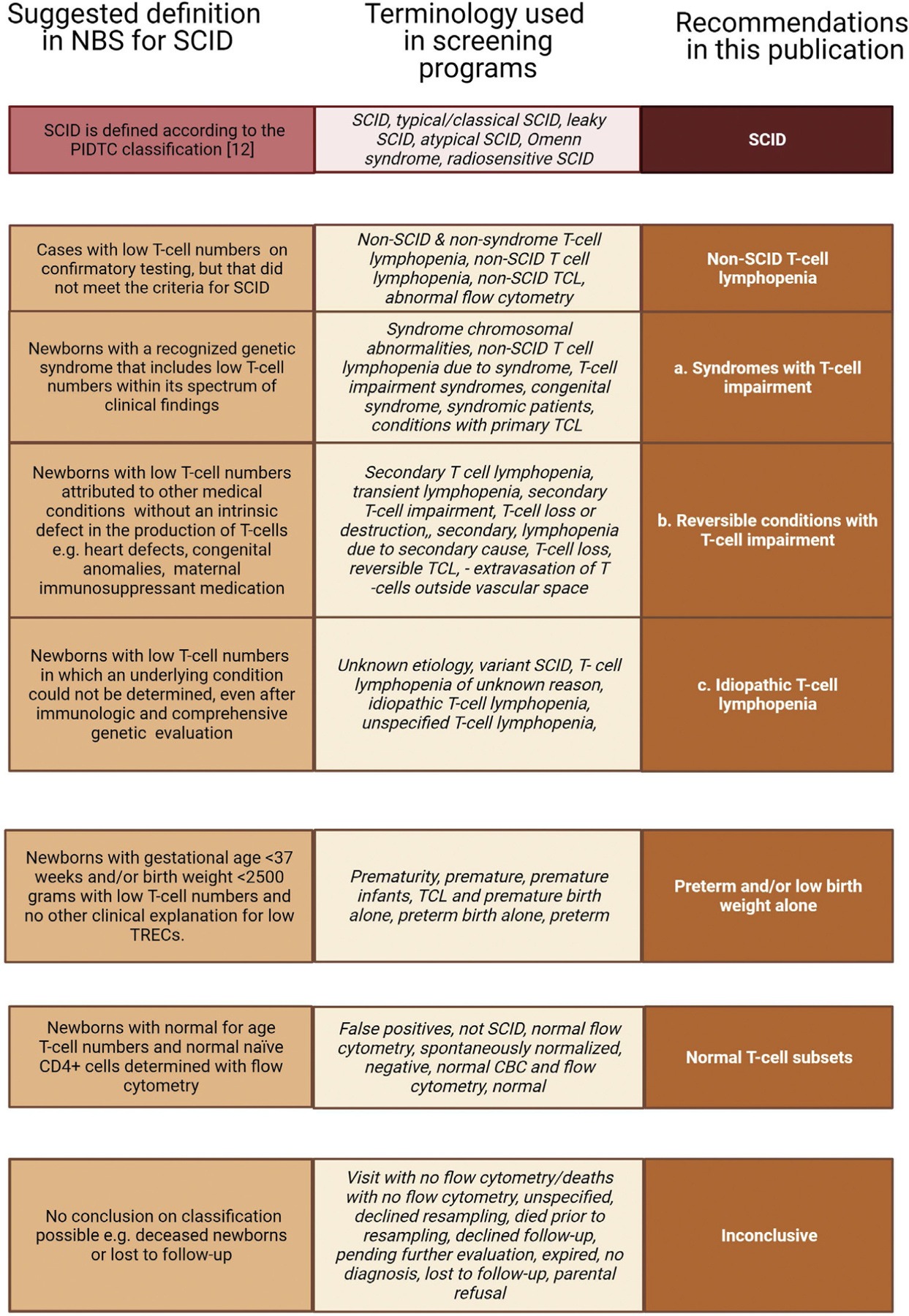
Recommendation on classification of diagnostic outcomes after an abnormal value screening test. *CBC*, Complete blood count; *TCL*, T-cell lymphopenia.

Preterms and/or newborns with low birth weight should be a separate category, only including preterm infants (gestational age <37 weeks) and/or newborns with low birth weight (<2500 g) who have low T cells without other preexisting conditions associated with T-cell lymphopenia. The term *false positive* can lead to confusion as some NBS programs define all referrals, with a diagnosis other than the disorder primarily screened for, SCID, as false-positives referrals. In addition, T cells may have been low at birth but normalized in the first week up to referral, reflecting a true transient T-cell lymphopenia. The term *normal T-cell subsets* is therefore better suited to avoid confusion. Finally, a subcategory was added to address *inconclusive* classification for newborns who have died prior to follow-up diagnostics or who are lost to follow-up without referral. Our recommendations will help with systematic registration of referred newborns and allow evaluation of NBS programs in a broader international perspective (Fig 4).

### Actionable T-cell lymphopenia versus nonactionable T-cell lymphopenia and secondary findings

An important aspect of TREC screening for SCID is the wide spectrum of different disorders that are detected by this single parameter. The TREC assay for SCID confers a high sensitivity compared to many established NBS disorders.^57^ In contrast, if one includes only SCID as the primary target of screening, the positive predictive value (PPV) is quite low as compared to some other screened disorders. NBS for SCID by quantification of TRECs identifies a range of neonatal conditions and disorders associated with T-cell lymphopenia in the neonatal period, which some programs define as secondary or incidental findings. NBS with TREC testing correlates with having recently formed T cells in peripheral blood; therefore one could argue that in TREC-based screening primary targets should include all serious, actionable T-cell deficiencies. From the clinical immunologist’s point of view, any newborn with a disorder in which prompt intervention can prevent morbidity and mortality should be flagged in a NBS program. NBS programs tend to focus on a primary target, although secondary targets/findings might be defined if there is a clear health benefit for the child. Policies differ between countries and individual screening programs in classifying severe T-cell deficiencies as primary or secondary targets (or findings) of NBS for SCID, and each NBS program will need to reach its own decision in this multifaceted discussion.

We feel that a distinction should be made between *actionable* and *nonactionable* T-cell lymphopenia and secondary findings, although it can be challenging to make clear statements about actionability. From a parental perspective, the benefit for actionable disorders lies in the possibility of managing the disease on recognizing it early in an infant’s life, thus improving health and social outcomes.^58^ Even in the absence of a cure, early diagnosis may lead to strategies resulting in health benefits such as prevention of comorbidities, facilitated access to social care and support, and improved quality of life. Parents also address the avoidance of a diagnostic odyssey and the option to make informed reproductive choices as clear health benefits, but we will limit the definition of actionability to the management of the individual affected with the condition. The term *actionable* indicates that an urgent (early) intervention is required by a specialist and that the intervention results in a demonstrated improvement in outcome. Neonates with profound T-cell lymphopenia, not meeting all criteria for SCID but eligible for HSCT, would undisputedly be classified as an actionable finding. The same would be applicable for patients with complete 22q11.2 deletion syndrome (DiGeorge syndrome), CHARGE (coloboma, heart defect, atresia choanae, restricted growth and development, genital abnormality, and ear abnormality) syndrome, athymic FOXN1 deficiency or PAX1 deficiency, all of which are indications for thymus transplantation.^59–62^ Pediatric immunologists propose that cases of significant T-cell lymphopenia that might benefit from antibiotic prophylaxis, protective isolation, or avoiding live-attenuated vaccines should also be deemed actionable.^63,64^ For these cases, one could argue that the term *actionable* depends on absolute T-cell number and the duration of the T-cell defect. The term *actionable* is more suitable than the term *treatable*, as withholding live-attenuated vaccines is an important early intervention leading to improved outcomes, given that vaccine-strain organisms can cause serious infections in individuals with T-cell defects.

Nonactionabl*e* secondary findings may be relevant prognostically, but either effective treatments are not available or health benefits from early diagnosis are limited or uncertain. The aim of population-based screening is to prevent morbidity or mortality from the targeted disorders through earlier treatment and with limited harm to unaffected infants. Nonactionable secondary findings and referrals of infants with normal lymphocyte numbers by flow cytometry raise concerns about the harm-benefit ratio of screening, and public health programs justifiably strive to prevent referral of these cases.^65^

### Defining targets for other conditions for which NBS is taking place

By better defining disease targets in a NBS program, parameters such as sensitivity/specificity and PPV can be reported and compared across programs, improving existing programs, but also aiding in policy with regard to pilot studies. NBS is a multifaceted system, and pilot studies provide the opportunity to consider addition of new disorders without disrupting the program. However, for smaller countries and in the case of rare diseases, pilot studies would require many years to generate data about sensitivity or PPV. If screening outcomes can be uniformly interpreted across borders, smaller countries might rely on test validation in screening laboratories and limit pilot studies to unique aspects of their locale. At this point, knowledge gained by other countries is not optimally used. If we would do so, swift implementation of new disorders could be achieved, saving time and money and leading to the most health gain for affected newborns. We suggest that international experts from each discipline included in NBS (eg, inborn errors of metabolism, congenital hypothyroidism, cystic fibrosis, hemoglobinopathies) join forces to discuss the target definitions and to provide their own recommendations for uniform registration of outcomes.

### The importance of uniform registration of screening outcomes

Public health programs have a responsibility toward their stakeholders to continuously improve and optimize their NBS programs. Opportunities for improvement can be identified only if outcomes can be compared with those of unscreened populations or other NBS programs. For international shared learning, harmonized registration of screening terminology and case definitions is a prerequisite. Evaluation of the screening terminology should be an ongoing process for continuous optimization of NBS programs. Trust in population screening programs is one of the key elements for parents when participating in NBS. By continuously optimizing laboratory algorithms and screening programs, increasing the PPV, one can limit the risk of unnecessary referrals that are associated with high emotional impact for parents and invasive diagnostic testing for the child.^32,65,66^ More importantly, an NBS program should aim to achieve the highest sensitivity, avoiding missing affected children in the direct health interest of the child. In addition, public health programs have a responsibility toward the society as a whole, as screening requires resources, and referrals are associated with high diagnostic costs. Cost-effectiveness analyses that are needed to justify NBS programs can be well-executed only if screening outcomes are registered in a uniform manner.

### Conclusions

Our recommendations reflect currently available evidence including a systematic literature review and existing guidelines coupled with expert opinion. By bringing two audiences together, the NBS community and the clinical immunology community, our guidelines will unite the field by bridging the gaps in language and perspective between these disciplines. Standardization of terminology and uniform registration of screening outcomes will promote international exchange of knowledge and improve NBS programs and follow-up care, resulting in better health outcomes for children worldwide.

## Supplementary Material

Supplementary Material (2)

Supplementary Material (1)

## Clinical implications:

Our recommendations reflect currently available evidence coupled with expert opinion for uniform registration of screening test outcomes and case definitions after diagnostic follow-up in NBS for SCID.

## Abbreviations used

APHL: Association of Public Health Laboratories
CLSI: Clinical and Laboratory Standards Institute
DBS: Dried blood spot
ESID: European Society for Immunodeficiencies
HSCT: Hematopoietic stem cell transplantation
IDF: Immune Deficiency Foundation
IEI: Inborn errors of immunity
IUIS: International Union of Immunological Societies
NBS: Newborn screening
PIDTC: Primary Immune Deficiency Treatment Consortium
PPV: Positive predictive value
SCID: Severe combined immunodeficiency
TREC: T-cell receptor excision circle
