# Supplementary material for: Recommendations for uniform definitions used in newborn screening for severe combined immunodeficiency
Source: J Allergy Clin Immunol. Author manuscript; Available in PMC 2023 Apr 1. (PMC9278646; doi:10.1016/j.jaci.2021.08.026)
Supplement: Supplementary Material (2) [file NIHMS1810523-supplement-Supplementary_Material__2_.pdf]

Online Repository Text

Methods

Systematic review

A systematic review was conducted on NBS for SCID and (case) definitions used in pilot studies and population based screening. The findings were reported according to the principles outlined in Preferred Reporting Items for Systematic Reviews and Meta-Analyses (PRISMA) statement (www. prisma-statement.org).

Literature search

An electronic search was performed on MEDLINE (PubMed), EMBASE (excluding MEDLINE), Cochrane library and Scopus databases on 16 February 2020. The keywords included synonyms for 'newborn screening' and 'primary immunodeficiencies/severe combined immunodeficiency/inborn errors of immunity'. Checking the reference lists of included studies identified additional publications. The search strategy is shown below in the Search Strategy.

Eligibility criteria

The initial search strategy was aimed at identification of all studies using definitions in NBS for SCID not discriminating between larger cohort studies and case studies. Systematic reviews, meta-analyses, randomized control trials, case reports, case-control studies, cohorts studies, letters to the editor without language restrictions were included in the initial search strategy. Exclusion criteria included publication of editorials, commentaries, unpublished manuscripts, dissertations, books and book chapters, lectures and speeches.

Study selection/Screening for inclusion

The study selection flow diagram is shown in Figure 1. Titles and abstracts of all the records retrieved from the electronic database searches were screened in a standardized manner. Records that were not clearly related to NBS for SCID and records that did not meet the eligibility criteria were excluded. Eligible articles were assed based on full text. Reviews were only included if classifications or definitions differed from previous original published articles. Case series describing retrospective TREC analysis of specific groups of PID patients were excluded.

Data extraction

Items that were extracted from included articles were: author, year, study design, study location, population based screening/pilot study, used TREC-assay (in house or CE-IVD marked assays), using second tier test option after TREC analysis, TREC cut-off value, number of newborns screened, number of retest on initial NBS cards, number of repeat DBS requested and number of referrals for follow-up diagnostics. If studies used an anonymized study population, presumptive positives rates were assumed referral rates. In addition, definitions on screening results, definitions or classifications used in screening algorithms and classifications of outcomes/diagnoses after follow-up were collected. Finally, definitions of premature birth and adjustments for premature infants in screening algorithms were extracted. The final selection of publications was discussed with the panel.

Quality assessment

The reviewer examined reports for completeness of reporting on screening outcomes, screening algorithms and classifications after follow-up. In some cases, multiple records described the same screening population. If the used definitions were consistent between these records, the most recent record was included. If information was missing in one of both studies, both articles would be included. Given the descriptive nature of this review, results were not combined for meta-analysis and a formal statistical analysis was not performed.

Search strategy (conducted on February 16, 2021)

PubMed

#1 (28444 hits)

“Newborn screening”[tiab] OR “Neonatal screening”[tiab] OR (“Newborn”[tiab] AND “Screening”[tiab]) OR (“Neonatal”[tiab] AND “Screening”[tiab]) OR “Guthrie card”[tiab] OR “Guthrie test”[tiab] OR “Dried blood spot”[tiab] OR “Dried blood spots”[tiab] OR “T cell receptor excision circle”[tiab] OR “T cell receptor excision circles”[tiab] OR “T-cell receptor excision circle”[tiab] OR “T-cell receptor excision circles”[tiab] OR “TREC”[tiab] OR “TRECs”[tiab] OR “Kappa deleting recombination excision circle”[tiab] OR “Kappa deleting recombination excision circles”[tiab] OR “Kappa-deleting recombination excision circle”[tiab] OR “Kappa-deleting recombination excision circles”[tiab] OR “KREC”[tiab] OR “KRECs”[tiab] OR “Neonatal screening”[MeSH] OR “Dried Blood Spot Testing”[MeSH]

#2 (34356 hits)

“PID”[tiab] OR “primary immune deficiency”[tiab] OR “primary immune deficiencies”[tiab] OR “primary immunodeficiency”[tiab] OR “primary immunodeficiencies”[tiab] OR “SCID”[tiab] OR “Severe combined immunodeficiency”[tiab] OR “T cell lymphopenia”[tiab] OR “T cell lymphopenias”[tiab] OR “T-cell lymphopenia”[tiab] OR “T-cell lymphopenias”[tiab] OR “T cell deficiencies”[tiab] OR “T-cell deficiency”[tiab] OR “T-cell deficiencies”[tiab] OR “T cell deficiency”[tiab] OR “inborn errors of immunity”[tiab] OR “Severe Combined Immunodeficiency”[MeSH] OR "Primary Immunodeficiency Diseases"[Mesh:NoExp]

#3 (515 hits)

#1 AND #2

EMBASE

#1 (10400 hits) AND [embase]/lim NOT ([embase]/lim AND [medline]/lim)

‘Newborn screening’:ti,ab OR ‘Neonatal screening’:ti,ab OR ‘Guthrie card’:ti,ab OR ‘Guthrie test’:ti,ab OR ‘Dried blood spot’:ti,ab OR ‘Dried blood spots’:ti,ab OR ‘T cell receptor excision circle assay’:ti,ab OR ‘T cell receptor excision circles’:ti,ab OR ‘T-cell receptor excision circle assay’:ti,ab OR ‘T-cell receptor excision circles’:ti,ab OR TREC:ti,ab OR TRECs:ti,ab OR ‘Kappa deleting recombination excision circle’:ti,ab OR ‘Kappa deleting recombination excision circles’:ti,ab OR ‘Kappa-deleting recombination excision circle’:ti,ab OR ‘Kappa-deleting recombination excision circles’:ti,ab OR #13 AND [embase]/lim NOT ([embase]/lim AND [medline]/lim)KREC:ti,ab OR KRECs:ti,ab OR ‘Newborn screening’/exp OR ‘Dried Blood Spot Testing’/exp

#2 (27614) AND [embase]/lim NOT ([embase]/lim AND [medline]/lim)

pid:ab,ti OR 'primary immune deficiency':ab,ti OR 'primary immune deficiencies':ab,ti OR 'primary immunodeficiency':ab,ti OR 'primary immunodeficiencies':ab,ti OR scid:ab,ti OR 'severe combined immunodeficiency':ab,ti OR 't cell lymphopenia':ab,ti OR 't cell lymphopenias':ab,ti OR 't-cell lymphopenia':ab,ti OR 't-cell lymphopenias':ab,ti OR 't cell deficiencies':ab,ti OR 't-cell deficiency':ab,ti OR 't-cell deficiencies':ab,ti OR 't cell deficiency':ab,ti OR 'inborn errors of immunity':ab,ti OR 'severe combined immunodeficiency'/exp OR 'primary immunodeficiency diseases'/mj

#3 (533 hits)

#1 AND #2

#4 (110 hits)

#3 AND (‘article’/it OR ‘article in press’/it OR ‘conference review’/it OR ‘editorial’/it OR ‘erratum’/it OR ‘letter’/it OR ‘note’/it OR ‘review’/it OR ‘short survey’/it)

Cochrane

#1 (1112 hits)

"Newborn screening" or "Neonatal screening" or "Guthrie card" or "Guthrie test" or "Dried blood spot" or "Dried blood spots" or "T cell receptor excision circle" or "T cell receptor excision circles" or "T-cell receptor excision circle" or "T-cell receptor excision circles" or "TREC" or "TRECs" or "Kappa deleting recombination excision circle" or "Kappa deleting recombination excision circles" or "Kappa-deleting

recombination excision circle" or "Kappa-deleting recombination excision circles" or "KREC" or "KRECs":ti,ab,kw

#2 (1688 hits)

"PID" or "primary immune deficiency" or "primary immune deficiencies" or "primary immunodeficiency" or "primary immunodeficiencies" or "SCID" or "Severe combined immunodeficiency" or "T cell lymphopenia" or "T cell lymphopenias" or "T-cell lymphopenia" or "T-cell lymphopenias" or "T-cell deficiency" or "T cell deficiency" or "T-cell deficiencies" or "T cell deficiencies":ti,ab,kw

#3 (8 hits)

#1 AND #2
