# Supplementary material for: Recommendations for uniform definitions used in newborn screening for severe combined immunodeficiency
Source: J Allergy Clin Immunol. Author manuscript; Available in PMC 2023 Apr 1. (PMC9278646; doi:10.1016/j.jaci.2021.08.026)
Supplement: Supplementary Material (1) [file NIHMS1810523-supplement-Supplementary_Material__1_.pdf]

Online Repository Tables

eTable 1. Study characteristics and details on included articles in the systematic review of definitions used in NSB for SCID

Author (year)	Ref	Study design	Location	Pilot study/ population based	Study Period	Parameter(s)<br>Assay<br>Cut-off value	Number screened<br>N	Number retest<br>N(%)	Number referrals<br>N (%)	Number of repeated DBS<br>N(%)
Thorsen et al. (2021)	(1)	Cohort	Wisconsin	Population based	January 2009 - December 2018	TREC<br>In house method<br>Cut-off: see	670,580<br>(91.7% full term)	NR	68 (0.01%)	NR
Richards et al. (2020)	(2)	Overview article	Australia and New Zealand	NA	NA	NA	NA	NA	NA	NA
Gizewska et al. (2020)	(3)	Cohort	Poland and Germany	Pilot study	October 2018 – December 2019	TREC/KREC<br>SPOT-it (ImmunoIVD)<br>TREC ≤ 6 copies/punch<br>KREC ≤ 4 copies/punch	44,287	321 (0.72%)	8 (0.02%)	168 inconclusive<br>58 second DBS positive<br>226 total 0.5%)
Blom et al. (2020)	(4)	Cohort	The Netherlands	Pilot study	April 2018 – February 2020	TREC<br>SPOT-it (ImmunoIVD)<br>TREC≤10 copies/punch	140,593	333 (0.24%)	47 (0.03%)	11 (0.01%)
Strand et al. (2020)	(5)	Cohort	Norway	Both	September 2015 - December 2017<br>January 2018 - August 2019	TREC<br>In house method<br>TREC ≤ 25/μL on first punch. After retest, second tier NGS for TREC 5-20/μL and ≤5/μL reported without delay	Pilot study 21,232<br>National implementation 88,000	37 (0.17%)<br>81 (0.09%)	4 reported (0.02%)<br>5 (0.006%) reported<br>1 contact NICU	13 (0.06%) / 12 received<br>21 (0.02%) / 11 received

Argudo-Ramírez et al. (2019)	(6)	Cohort	Catalonia (Spain)	Population based	January 2017 - December 2018	TREC<br>EnLite (PerkinElmer)<br>2017: TREC ≤34 copies/µL<br>2018: TREC ≤24 copies/µL<br>After retest: TREC≤20 copies/µl	129,614	3108 (2.4%)	30 (0.02%)	304 (0.2%)
Thomas et al. (2019)<br>Audrain et al. (2018)	(7, 8)	Cohort	France	Pilot study	January 2015 – March 2017	TREC<br>EnLite (PerkinElmer)<br>TREC ≤35 copies/µL on first punch. After retest, TREC ≤10 copies/µl for referral or 11-21 for second DBS	190,517	5106 (2.68%)	165 (0.087%)	291 (0.15%)
Amatuni et al. (2019)	(9)	Cohort	California	Population based	August 2010 - May 2017	TREC<br>In house TREC ≤25 copies/µl 2010-2015<br>Enlite PE TREC≤ 18 copies/µl from June 2015	3,252,156	NR	562 (0.02%)	NR
Kobrynski et al. (2019)	(10)	Overview article	USA	NA	NA	NA	NA	NA	NA	NA
Nourizadeh et al. (2018)	(11)	Cohort	Iran	Pilot study	Not specified	TREC/KREC<br>Early version ImmunoIVD-assay<br>TREC <11 copies/punch<br>KREC <6 copies/punch	2,160	30 positive (1.4%)<br>(21 retested)<br>168 (7.8%) inconclusive (not retested)	3 retested (0.15%)<br>9 non-repeated (0.45%)	No access to infants: 168 inconclusive (7.8%)
Al-Mousa et al. (2018)	(12)	Cohort	Saudi Arabia	Pilot study	November 2015 – November 2016	TREC<br>EnLite (PerkinElmer)<br>TREC <36 copies/µL<br>Second tier: targeted NGS PID panel	8,718	315 (3.6%)	16 (0.18%)	No further testing: 70 inconclusive (0.8%)
Routes, Verbsy et al. (2018)	(13)	Overview article	USA Wisconsin	NA	NA	NA	NA	NA	NA	NA
Rechavi et al. (2017)	(14, 15)	Cohort	Israel	Population based	October 2015-September 2019	TREC<br>EnLite (PerkinElmer)<br>TREC <36 copies/blood spot lowered to 23 copies/blood	177,277	*36*: 7517 (4.24%)<br>*28*: 3262 (1.84%)	46 (0.02%)	561 (0.3%)

						spot. Second Guthrie card for confirmation		*23*: 1684 (0.95%)		
Son et al. (2017)	(16)	Cohort	Korea	Pilot study	August 2015 – December 2015	TREC/KREC<br>In house method<br>Cp value < 37.0 TREC/KREC positive, Cp value >37.0 and <39.0 TREC/KREC weak positive, Cp value >39.0 TREC/KREC negative	141	1 (0.7%)	1 (0.7%) weak positive	NR
Kanegae et al. (2017)	(17)	Cohort	Brazil	Pilot study	September 2014 - July 2015	TREC/KREC<br>In house method<br>Initial: 25 TRECs/KRECs/µL, adjusted to 15 TRECs/µL and 14 KRECs/µL	6,881	*25*: 172 (2.5%)<br>*15/14*: 34 (0.49%)	2 (029%)	NR
Kanegae et al. (2016)	(18)	Cohort	Brazil	Pilot study	Not specified	TREC<br>In house method<br>Cut-off: 30 TRECs/µL<br>Cut-off: 26 TRECs/µL	8,682	*30*: 49 (0.56%)<br>*26*: 37 (0.43%)	4 (0.05%)<br>3 (0.03%)	0
Tagliaferri et al. (2017)	(19)	Cohort	Germany	Pilot study	October 2012 - December 2012	TREC<br>In house method<br><95 TREC copies/1.6 mm DBS punch.	6,034	70 (1.2%)	3 (0.05%)	NR
Barbaro et al. (2017)	(20)	Cohort	Sweden	Pilot study	November 2013 - November 2015	TREC/KREC<br>Early version ImmunoIVD assay<br>Rerun cut-off: TREC 25/ KREC 15 copies/punch<br>Three recall cut-off values used:<br>TREC 15/ KREC 10<br>TREC 8/ KREC 4<br>TREC 10 / KREC 6	58,834	572 (0.97%)<br><br>259 for only TREC and inconclusive samples (0.44%)	64 (0.11%)<br><br>16 for TREC only (0.03%)	13 (0.02%)
Zetterström et al. (2017)	(21)	Cohort	Sweden	Pilot study	November 2013 - November 2016	TREC/KREC<br>Early version ImmunoIVD assay<br>Cut-off values see (20)	89,462	972 (1.09%)<br><br>418 for only TREC and inconclusive samples (0.47%)	93 (0.10%)<br><br>25 for TREC only (0.03%)	15 (0.02%)

De Felipe et al. (2017)	(22)	Cohort	Spain	Pilot study	February 2014 - December 2016	TREC/KREC<br>Early version ImmunoIVD assay<br>TRECs < 6/punch, ACTB > 700/punch and KRECs < 4/punch	8,943	124 (1.39%)	5 (0.055%)	10 (0.11%)
De Felipe et al. (2015)	(23)	Cohort	Spain	Pilot study	February 2014 - March 2015	TREC/KREC<br>Early version ImmunoIVD assay<br>Cut-off values see (22)	5,160	109 (2.11%) but 77 (1.5%) retested due to insufficient material	5 (0.1%)	10 (0.19%)
Blom et al. (2017)	(24)	Cohort	The Netherlands	Pilot study	Not specified	TREC<br>EnLite (PerkinElmer)<br>TREC <40 copies/µL<br>TREC <22 copies/µL	1,295	*40*: 39 (3.0%)<br>*22*: 2 (0.15%)	*40*: 21 (1.62%)<br>*22*: 1 (0.08%)	1 (0.08%)
Chien et al. (2017)	(25)	Cohort	Taiwan	Population based	May 2010-June 2016	**NTUH**: In house, <40 TRECs/µL<br>**CFOH**: In house, Different cutoffs for the 1st/2nd DBS samples (initially 35/30 copies/µL, revised to 50/40 copies/µL September 2015)<br>**TIP**: EnLite (PE) TREC <25 copies/µL	920,398	NR	175 (0.02%)	NR
Madkaikar et al. (2016)	(26)	Overview article	India	NA	NA	NA	NA	NA	NA	NA
Chien et al. (2015)	(27)	Cohort	Taiwan	Pilot study	May 2010 - December 2011	TREC<br>In house<br><40 TRECs/µL,	106,391	NR	24 (0.02%)	432 (0.4%)
Kwan et al. (2015)	(28)	Cohort	Navajo Nation	Both<br>Pilot study	March 2009 - February 2012	TREC<br>In house method<br>TREC <33/uL	1,800	NR<br>(less or equal 13)	2 (0.1%)	11 (0.6%)
				Population based	Not specified	EnLite (PerkinElmer)<br>TREC < 40 copies/µL<br>TREC < 25 copies/µL	3,733	2 (0.05%)	2 (0.05%)	0

Kwan et al. (2014)	(29)	Cohort	USA	Both	2008 - 2013	TREC<br>Different methods with different cut-off values see Supplement in (29)	3,030,083	NR	1,295 (0.04%)	NR
Audrain et al. (2014)	(30)	Cohort	France	Pilot study	June 2012-October 2012	TREC<br>In house method<br>TREC>183 copies/reaction<br>TREC>100 copies/reaction	5,028	*183*: 132 (2.6%)<br>*100*: 59 (2.0%)	NR (but recall for equivocal)<br><br>*183*: 2 (0.04%)<br>*100*: 0	NR (but recall for inconclusive)<br>*183*: 9 (0.18%)<br>*100*: 2 (0.04%)
Adams et al. (2014)	(31)	Cohort	UK	Pilot study	Not specified	TREC<br>EnLite (PerkinElmer)<br>TREC<40 copies/uL<br>TREC<20 copies/uL	5,081	*40*: 191 (3.76%)<br>*20*: 10 (0.20%)	*40*: 51 (1.00%)<br>*20:* 2 (0.04%)	*40*: 1 (0.02%)
Vogel et al. (2014)	(32)	Cohort	New York	Population based	September 2010 - September 2012	TREC<br>In house method<br>≤200 TRECs	485,912	NR	531 (0.11%)	561 (0.12%) premature<br>746 (0.15%) non-premature
Kwan et al. (2013)	(33)	Cohort	California	Population based	2012-2013	TREC<br>In house method<br>< 40 TRECs/µL<br>Second cut-off <25 TRECs/µL	993,724	NR	161 (0.02%)	806 (0.08%)
Borte et al. (2012)	(34)	Cohort	Sweden	Pilot study	Not specified	TREC/KREC<br>Early version Immuno-IVD assay<br>TREC <15/uL<br>KREC< 10/uL	2560	32 (1.25%)	6 (0.23%)	1 (0.04%)

Verbsky et al. (2011)	(35)	Cohort	Wisconsin	Population based	January 2008 - December 2010	TREC<br>In house method<br><br>TREC <25, but increased to 40 in August 2009.	207,696	NR	63 (0.03%)<br><br>(72 in the end)	0.19%<br><br>51 inconclusive full terms<br>94 abnormal preterms<br>241 inconclusive preterms
Comeau et al. (2010)	(36)	Cohort	Massachusetts	Pilot study	February 2009 - January 2010	TREC<br>In house method<br>Cut-off value not specified	76,843	3842 (5%)	350 (0.45%)<br><br>51 referred for diagnostic evaluation (0.07%)	181 (0.24%)
Baker et al. (2010)<br><br>Routes et al. (2009)	(37, 38)	Cohort	Wisconsin	Population based	2008	TREC<br>In house method<br><br><25 TRECs/μL	71,000	NR	12 abnormal full term (0.02%)<br><br>(11 abnormal for flow cytometry evaluation)	23 inconclusive full terms (0.03%)<br>23 abnormal preterms (0.03%)<br>96 inconclusive preterms (0.14%)

*NR not reported, NA not applicable

eTable 2. Terminology and definitions of screening results used in studies on NBS for SCID

Author (year)	Ref	Location	Terminology used	Definition
Thorsen et al. (2021)	(1)	Wisconsin	Screen positive/ positive NBS SCID screen<br>Abnormal NBS SCID	Specified in previous publications (35, 37, 38)
Richards et al. (2020)	(2)	Australia and New Zealand	Presumptive positive<br>Indeterminate / inconclusive	TREC < cut-off on duplicate testing, normal internal control on duplicate testing<br>Low TREC (may be <cut-off), low or absent internal control on duplicate testing
Gizewska et al. (2020)	(3)	Poland and Germany	Incomplete<br>Negative/normal<br>Positive<br>Urgent positive<br>Inconclusive<br>Abnormal	Sample TREC < cut off needs retest of same sample in duplicate<br>TREC levels > cut-off after initial analysis or repeated analysis in duplicate<br>TREC ≤ cut-off and ACTB ≥ 1000 copies/punch after repeated analysis in duplicate<br>TREC < 1 copies/punch from the first blood samples (3 DBS punches)<br>TREC ≤ cut-off and ACTB < 1000 copies/punch after repeated analysis in duplicate<br>ACTB >1,000 copies/μL and TREC copy numbers below the respective cutoff value
Blom et al. (2020)	(4)	The Netherlands	Negative<br>Positive/abnormal<br>Inconclusive	TREC levels > cut-off after initial analysis or repeated analysis in duplicate<br>TREC ≤ cut-off and ACTB ≥ 1000 copies/punch after repeated analysis in duplicate<br>TREC ≤ cut-off and ACTB < 1000 copies/punch after repeated analysis in duplicate
Strand et al. (2020)	(5)	Norway	Abnormal<br>Normal<br><br>Inconclusive<br>Normalized<br>Screening positive	Mean TREC <25/uL and beta-actin >5000/uL after repeated testing<br>TREC > 25/uL after first analysis or normalized after TREC>25/uL and beta-actin ≥ 5000/uL after repeated analysis<br>Beta-actin <5000/uL after repeated testing<br>After repeated analysis TREC>25/uL and beta-actin ≥5000<br>Samples with the lowest TRECs (≤ 5/μL) are screening positive. For intermediate low TRECs (5–20/μl) only those with molecular confirmation of disease (defined as ACMG class 4 or 5, or a class 3 variant in trans with a 4–5) (32), were regarded as screening positives
Argudo-Ramírez et al. (2019)	(6)	Catalonia (Spain)	Negative detection or normal/negative result<br>Positive detection	TREC levels >cut-off (24) after initial analysis or repeated analysis (2/3 or second sample collection<br>Samples with TRECs ≤ 5 copies/μL (preterm infants) or ≤ 10 copies/μL (term newborns) in the first sample (both with beta-actin gene ≥50 copies/μL), as well as analyses with TRECs ≤ 20 copies/μL in the second sample: referred to SCID CRU
Thomas et al. (2019)<br>Audrain et al. (2018)	(7, 8)	France	Negative<br>Inconclusive<br><br>Positive	>34 copies/μL on initial test or 2 out of 3 values >20 copies/μL after retest<br>2 out of 3 values between 10-21 for full term babies and 5-21 for preterm babies or <21 and no actin amplification<br>2 out 3 values (copies/μL) <11 for full-term babies or <5 for preterm babies

Amatuni et al. (2019)	(9)	California	Normal<br>Urgent positive<br><br>Positive<br><br>Incomplete	TREC >18a: N/Ab, no further action<br>TREC Undetectable or <4c >35d, immediate callback for liquid blood for lymphocyte subsets<br>TREC 4–18, infant not in NICU >35, positive, callback for liquid blood for lymphocyte subsets<br>TREC 4–18, infant in NICU N/A, incomplete, second DBS test in 2 wk or at discharge from nursery; after 2 incomplete test results, liquid blood for lymphocyte subsets
Kobrynski et al. (2019)	(10)	USA	Abnormal SCID NBS	TREC below cut-off value
Nourizadeh et al. (2018)	(11)	Iran	Abnormal / Positive<br>Normal<br>Inconclusive	ACTB ≥700/uL, TREC or KREC < cut-off value<br>ACTB>700, TREC and KREC ≥ cut-off<br>ACTB <700/uL and TREC or KREC < cut-off
Al-Mousa et al. (2018)	(12)	Saudi Arabia	Normal<br>Abnormal for retest<br>Inconclusive	TREC>36 copies/uL and beta-actin ≥56 copies/uL<br>Initial TREC or beta-actin copy value below the cutoff<br>TREC <36 copies/uL and beta-actin <56 copies/uL (2 repeated samples)
Routes, Verbsy et al. (2018)	(13)	USA Wisconsin	Abnormal TREC screen	Specified in previous publications [33-35]
Rechavi et al. (2017)	(14, 15)	Israel	Positive/abnormal<br>Initial positives<br>True positives<br><br>Negative NBS result	TREC < cut-off<br>TREC < cut-off after initial measurement<br>TREC < cut-off in five measurements (after retesting same Guthrie card, second Guthrie card for validation/confirmation)<br>TREC > cut-off
Son et al. (2017)	(16)	Korea	TREC/KREC positive<br>TREC/KREC weak positive<br>TREC/KREC negative	A Cp value less than 37.0 was defined as TREC/KREC positive<br>A Cp value more than 37.0 and less than 39.0 was defined as TREC/KREC weak positive<br>A Cp value more than 39.0 was defined as TREC/KREC negative
Kanegae et al. (2017)	(17)	Brazil	Normal<br>No other definitions used	TRECs and KRECs > 25 copies/uL (within normal parameters)
Kanegae et al. (2016)	(18)	Brazil	Normal<br><br>Abnormal<br><br>Inconclusive	A cutoff value of 30 TRECs/μL of blood was arbitrarily used to determine whether a sample was normal<br>After the second analysis, the samples with values <30 TRECs/μL and beta-actin >8000/μL were considered abnormal<br>TRECs <30/μL and beta-actin <8000/μL were classified as inconclusive result and a new sample was requested
Tagliaferri et al. (2017)	(19)	Germany	Real TREC-negative results	Initial TREC value below the cutoff, TREC below cut-off in second tier and beta-actin above cut-off in second tier

			DNA amplification failure	Initial TREC value below the cutoff, TREC below cut-off in second tier/beta-actin below cut-off in second tier
Barbaro et al. (2017)	(20)	Sweden	Abnormal/positive<br><br>Inconclusive<br><br>Normal	Samples with TREC and/or KREC copies below the cutoff values were considered “abnormal” (positive),<br>Samples in which TREC and/or KREC levels were below cutoff in association with a reduction in ACTB copy number ≤1000 copies/punch were considered inconclusive<br>TREC and KREC above cutoff values
Zetterström et al. (2017)	(21)	Sweden	Abnormal/positive<br><br>True positive<br>Inconclusive/Inadequate<br><br><br><br><br>Normal	Samples with TREC and/or KREC copies below the cutoff values were considered “abnormal” (positive)<br>Confirmed immune deficiency<br>ACTB <1000 copies per punch and KREC and/or TREC copies below repeat test cut-off values, were reanalyzed. If still inconclusive, a punch from each blood spot (usually four) on the sample was analyzed. If a sample was still inconclusive after the repeat testing, it was considered inadequate and a new sample was requested<br>TREC and KREC above cutoff values
De Felipe et al. (2017)<br>De Felipe et al. (2016)	(22, 23)	Spain	Pathological results/positive results/abnormal results<br>I<br>nconclusive<br>Normal results	Abnormal or inconclusive results required a new punch from the same DBS and a repeat PCR-assay (re-test) was performed. Subsequently, a pathological result in the re-test required a new heel prick sample (re-sample), and the confirmation of a result below the established cut-off resulted in a physical assessment of the neonate in the immunology clinic. If the material of the 1st DBS was insufficient, re-punching was performed (re-call) with extraction of a 2nd DBS (re-sample).
Blom et al. (2017)	(24)	The Netherlands	Normal<br><br>Positive<br>Presumptive positive<br><br>Inconclusive	TREC ≥40 copies/uL after initial analysis or TREC ≥ 40 copies and beta-actin ≥ 40 copies/uL after repeated testing in duplicate<br>TREC <40 copies/uL after initial analysis<br>TREC < 40 copies in either duplicate and beta-actin ≥ 40 copies/uL after repeated testing in duplicate<br>Beta-actin < 40 copies after repeated testing in duplicate
Chien et al. (2017)	(25)	Taiwan	Abnormal SCID screen result	Abnormal TREC copy numbers. See Chien et al. (2015) (27)
Madkaikar et al. (2016)	(26)	India	Abnormal<br>Normal<br><br>Inconclusive	TREC< normal cut off and house keeping gene normal after repeat qPCR<br>TREC > normal cut-off or TREC > normal cut off and house keeping gene normal after repeat qPCR<br>If the reference gene is not detected, the test is termed inconclusive
Chien et al. (2015)	(27)	Taiwan	Abnormal<br>Inconclusive	A DBS with a zero TREC value but a normal RNase P value.<br>A DBS with a TREC value between zero and 40, all inconclusive DBSs required a

			Normal	repeat DBS, and either a low or zero TREC value on the repeat DBS was defined as abnormal<br>A DBS with a TREC value >40
Kwan et al. (2015)	(28)	Navajo Nation	Normal<br><br>Inconclusive<br><br>Positive<br>Presumptive positive	TREC>33 after 1st or repeat run / TREC>40 in initial DBS sample or TREC>25 after repeated analysis<br>Low TRECs and low beta-actin / TREC 0 and beta-actin <5000 or TREC 1-25 and beta-actin <10000<br>Low TREC (<33) and normal beta-actin / TREC = 0 and beta-actin>5000<br>TREC 1-25 and beta-actin > 10000
Kwan et al. (2014)	(29)	USA	Kwan et al: "A major limitation of this study was the lack of uniformity of assay methodology and rules for retesting among the individual newborn screening programs. Use of different TREC assays and test algorithms resulted in a variety of rates both for recall for additional testing and for having T cells by flow cytometry in a range defined as normal"	
Audrain et al. (2014)	(30)	France	Normal<br>Abnormal<br>Equivocal<br>Inconclusive	Above 183 TREC copies/reaction<br>Fewer than 39 TREC copies/reaction<br>Between 39 and 183 TREC copies/reaction with RNAseP amplification<br>Fewer than 183 TREC copies/reaction and no RNAseP amplification.
Adams et al. (2014)	(31)	UK	Negative/Normal negative<br><br>Invalid result<br>Presumptive positive result	Initial TREC testing singlicate ≥ cut-off or TREC ≥ cut-off in both duplicates after repeat testing with new punches and beta-actin duplicates ≥cut-off<br><br>Either beta-actin duplicate below cut-off after repeat testing<br>TREC < cut-off in either duplicate and both beta-actin duplicates ≥ cut-off
Vogel et al. (2014)	(32)	New York	Screen negative<br><br>Abnormal<br><br>Borderline/presumptive positive<br>Borderline for preterms<br><br>Screen positive<br><br>Inconclusive	Samples with >200 TRECs and an RnaseP Cq value <35 were considered to be within acceptable limits (screen negative)<br>Samples with ≤200 TRECs and/or an RnaseP Cq value <35 were considered abnormal and required repeated testing in duplicat<br>Samples with ≥125 TRECs/uL and ≤200 TRECs and RnaseP Cq value <35 and gestational age ≥37 weeks<br>Samples with ≤200 TRECs and/or an RnaseP Cq value <35 and gestational age <37 weeks: repeat requested when GA ≥37 weeks<br>TRECs = 0 and RnaseP Cq value <35 for all gestational ages or <125 TRECs/uL and RnaseP Cq value <35 and gestational age ≥37 weeks<br>Pending diagnostic testing or lost to follow-up
Kwan et al. (2013)	(33)	California	Normal<br>Positive<br>Urgent positive	Samples with more than 40 TRECs/μL on initial testing were considered normal<br>Initial TREC value below the acceptable cutoff

			 <br>DNA amplification failure<br>Incomplete	Those with undetectable or 1–5 TRECs/μL of blood with normal control β-Actin copies >5000 copies<br>Initial samples with low TRECs, but also low β-Actin<br><br>NICU screen result with 6 to 25 TRECs/μL and a β-actin copy number of 10,000 or less.
Borte et al. (2012)	(34)	Sweden	Normal<br><br>Inconclusive<br><br>Abnormal	ACTB ≥1000 copies, TREC≥15/uL and KREC ≥10 after initial testing or after repeated testing<br>ACTB copy numbers below 1000/μL and concomitant reduction of TRECs and KRECs were referred to as “inconclusive”<br>TREC or KREC copy numbers below the respective cutoff values both after initial testing and repeated testing (are both called abnormal)
Verbsky et al. (2011)	(35)	Wisconsin	Abnormal<br><br><br>Inconclusive	TREC level below the cut-off were first tested for DNA integrity by analyzing β-actin by qRT-PCR. If the β-actin level was normal with abnormally low TRECs, abnormal report was issued for full term infants.<br>If the β-actin result was low, an inconclusive report was issued and the screening test was repeated with a new newborn screening card.
Comeau et al. (2010)	(36)	Massachusetts	Negative SCID NBS report<br>Positive SCID NBS report<br><br>Unsatisfactory SCID NBS report	TREC values indicating a number within the normal range for neonatal TREC copies/μl whole blood<br>A positive SCID NBS result is characterized by a low TREC value (two of the three TREC values were below cutoff) with a valid result for the internal control (RNaseP).<br>Specimens without amplifiable DNA (RNaseP values below cutoff on two of the three results) were considered unsatisfactory.
Baker et al. (2010)<br><br>Routes et al. (2009)	(37, 38)	Wisconsin	Abnormal or inconclusive<br><br><br>Normal	Specimens not reported as normal (i.e., ≥25 TRECs/μL) fall into one of two categories: inconclusive (TREC<25/uL with low beta-actin) or abnormal (TREC< 25/ul: with normal beta-actin)<br>Specimens with ≥25 TRECs/μL are considered to be normal (i.e., negative for SCID and other immunodeficiencies).

eTable 3. Terminology and definitions of variables in the screening algorithm used in studies on NBS for SCID

Author (year)	Ref	Location	Terminology used	Definition
Thorsen et al. (2021)	(1)	Wisconsin	Referral	Referred to one of the two centers for confirmatory testing and, if indicated, follow-up care
Richards et al. (2020)	(2)	Australia and New Zealand	Repeated testing	Following identification of low or absent TREC by NBS, testing will be repeated and second-tier laboratory testing will be required
Gizewska et al. (2020)	(3)	Poland and Germany	Retest<br>Second sample<br>Recall	Re-tested on the first DBS in duplicate<br>Repeated sampling, collection of a second blood sample<br>Recalled for further immunological evaluation/confirmatory diagnosis (either after re-testing 1st DBS or 2nd DBS)
Blom et al. (2020)	(4)	The Netherlands	Retest<br><br>Second DBS/Second NBS sample<br>Referral	Repeated TREC analysis in duplicate after initial TREC analysis on the same NBS card (two punches)<br>Repeated sampling: collection of a second newborn screening card<br>Referral for additional diagnostics
Strand et al. (2020)	(5)	Norway	Rerun<br><br>Repeated filer card sample / Second DBS sample / Redraw<br><br><br><br>Recall/referral	Samples below 25 TRECs/μL are re-punched and TREC analyses repeated twice on DNA from the new punch<br>A new DBS sample is requested if low levels of β-actin (<5,000/μL) are found. If TRECs are below 15/μL and NBS-NGS gene panel negative in an apparently healthy child with normal weight born to term, a second DBS sample is requested as a "safety net.<br>Admission and clinical follow-up in the hospital
Argudo-Ramírez et al. (2019)	(6)	Catalonia (Spain)	Retest<br>Second sample<br>Positive detection	Repetition of the same sample in duplicate<br>New DBS card<br>Positive detections were notified to the SCID Clinical Reference Unit (SCID-CRU) to initiate clinical and immunological evaluation
Thomas et al. (2019)<br>Audrain et al. (2018)	(7, 8)	France	Retest<br>Second DBS<br>Recall<br>Referred	Retest of the same sample in duplicate<br>New blood sample is collected<br>Recall for either a second DBS or for an appointment<br>Referred for diagnostic evaluation
Amatuni et al. (2019)	(9)	California	Retesting<br>Callback<br>Second DBS<br>Referral	Not mentioned, only for preterms on second DBS<br>Liquid blood for lymphocyte subsets<br>Second sample collection<br>To pediatric hospitals after abnormal flow cytometry results (first interpreted by immunology associates)

Kobrynski et al. (2019)	(10)	USA	NR	NR
Nourizadeh et al. (2018)	(11)	Iran	Retest<br>Second sample	A new punch of old Guthrie cards was taken and was analyzed<br>Re-testing of a second sample (not executed in the pilot study)
Al-Mousa et al. (2018)	(12)	Saudi Arabia	Retest / repeat testing<br>Referral / recall	Second (repeat) TREC analysis<br>Referrals for confirmatory studies /clinical and immunological evaluation
Routes, Verbsy et al. (2018)	(13)	USA Wisconsin	Repeated<br>Other definitions specified in previous publications [33-35]	TREC assays are repeated and not considered abnormal when both the numbers of TRECs and β-actin are low
Rechavi et al. (2017)	(14, 15)	Israel	Retest<br><br>Second Guthrie card<br><br>Referral	Retesting consists of two additional punches taken from different DBS of the same, initial Guthrie card<br>If both are below cut-off for TREC with normal amplification of beta-actin, a second, confirmation Guthrie card is obtained and tested in duplicate<br>Referral to the national center for SCID screening confirmation
Son et al. (2017)	(16)	Korea	Repeated testing	Not specified
Kanegae et al. (2017)	(17)	Brazil	Repetition rate/reanalysis<br><br>Referral	New DNA extraction, re-examined aggregating the beta-actin analysis for the extraction quality control<br>Referred to pediatric immunologist/allergist for evaluation and confirmatory testing
Kanegae et al. (2016)	(18)	Brazil	Second analysis/ repeated TREC analysis<br>New sample<br>Referred	New piece of the same sample had its TRECs analysis repeated accompanied by beta-actin analysis, as extraction control<br>Request of a new sample for inconclusive results<br>Referred to a pediatric immunologist for consultation and confirmatory tests
Tagliaferri et al. (2017)	(19)	Germany	Retest<br><br><br>Recall	Second TREC analysis including an internal control with beta actin (same punch, same DNA) If no beta-actin, second punch from the same blood sample.<br>Only samples that failed the second tier would be recalled in a non-anonymized setting.
Barbaro et al. (2017)	(20)	Sweden	Repeat testing<br>Recall (repeat sampling/resampling)<br>Recall (for follow-up/referred)	Repeat testing on the original DBS, reanalyzed in duplicate<br>New sample requested<br>Referred to a pediatrician specialized in the diagnosis and management of PID.

Zetterström et al. (2017)	(21)	Sweden	Repeating testing/ rerun<br><br><br>Repeat DBS/second sample<br>Recall (repeat sampling/resampling)<br>Recall (for follow-up/referred)	Reanalyzed in duplicate, taking a new punch from another blood spot on the filter paper sample. If still inconclusive a punch from each blood spot (usually four) on the sample was analyzed<br>New sample requested<br>Recalled for new sample<br>Recalled for clinical evaluation if the mean values of the three analyses of TREC and/or KREC were below recall levels and the simultaneously determined ACTB control was above 1000 copies per punch
De Felipe et al. (2017)<br>De Felipe et al. (2016)	(22, 23)	Spain	Retest<br>Re-sample<br>Re-call<br>The confirmation of a result below the established cut-off resulted in a physical assessment of the neonate in the immunology clinic	A new punch from the same DBS and a repeat PCR-assay (re-test)<br>A pathological result in the re-test required a new heel prick sample: re-sample. If the material of the 1st DBS was insufficient, re-punching was performed (re-call) with extraction of a 2nd DBS (re-sample)
Blom et al. (2017)	(24)	The Netherlands	Retest<br>Second heel prick<br>Referral	TREC analysis repeated in duplicate from the same heel prick card<br>Request of a second heel prick sample<br>Referral for confirmatory diagnostics
Chien et al. (2017)	(25)	Taiwan	Retest<br>2nd DBS request<br>Refer	Retest TREC measurement by another punch from the 1st DBS<br>Second dried blood spot (in case TREC >0-40 and normal Rnase P)<br>Referred for flow cytometry/ confirmatory immunological function evaluation
Madkaikar et al. (2016)	(26)	India	Repeat testing<br><br>Referred	Samples with the abnormal multiplex result are retested using the same multiplex assay<br>Referred to the specialized center with necessary expertise in the diagnosis and management of PIDs
Chien et al. (2015)	(27)	Taiwan	Repeat NBS/DBS<br>Refer	Second DBS sample<br>Referred for confirmatory tests
Kwan et al. (2015)	(28)	Navajo Nation	Second punch analyzed<br>Repeat TREC testing on second DBS<br>Refer	A second punch reanalyzed for TREC and β-actin copies<br>Second sample collection and repeating analysis<br>Samples with 2 poor PCR results or low TRECs with normal β-Actin were reported to the study workers for clinical evaluation
Kwan et al. (2014)	(29)	USA	Kwan et al: “A major limitation of this study was the lack of uniformity of assay methodology and rules for retesting among the individual newborn screening programs. Use of different TREC assays and test algorithms resulted in a variety of rates both for recall for additional testing and for having T cells by flow cytometry in a range defined as normal”	

Audrain et al. (2014)	(30)	France	Re-test / Second run<br><br><br>Recall	For equivocal or inconclusive results: the result was below cut-ff, a second punch from the same sample was re-extracted and a new RT-qPCR was performed.<br>Recalled not specified
Adams et al. (2014)	(31)	UK	Retesting<br><br>Repeat heel prick<br>Refer	Repeat tested on duplicate punches from the same DBS used for the initial punch<br>DNA amplification failure would require a second heel prick blood spot<br>Referral for confirmatory testing
Vogel et al. (2014)	(32)	New York	Retest in duplicate<br><br>Repeat sample<br>Referred	Retested in duplicate using a fresh DBS punch and a manual version of the same DNA extraction<br>Repeat NBS, repeat specimen<br>Referral for diagnostic evaluation
Kwan et al. (2013)	(33)	California	Repeat TREC test<br>Second DBS<br>Recall<br>Refer	Repeat TREC with β-actin testing<br>New heel-stick sample<br>Liquid blood sample for flow cytometry<br>Referral to PID center (if T-cells <1500 CD3+ T-cells or absent T-cells)
Borte et al. (2012)	(34)	Sweden	Repeat testing	Repeat testing was carried out using a second dried blood spot punch from the original/same Guthrie card.
Verbsky et al. (2011)	(35)	Wisconsin	Repeat testing<br>New newborn screening card<br>Referred	Screening test was repeated with a new newborn screening card<br>New sample collection<br>Infants with an abnormal flow cytometry were then referred for evaluation by a clinical immunologist
Comeau et al. (2010)	(36)	Massachusetts	Retest<br>Repeat NBS specimen<br>Referral	Re-tested in duplicate with new 3-mm punches from the same specimen<br>Repeat NBS specimen from the infant<br>Referral for diagnostic evaluation.
Baker et al. (2010)<br><br>Routes et al. (2009)	(37, 38)	Wisconsin	Retested<br><br>New NBS card/second NBS<br><br>Follow-up	Second round of analysis for TRECs and β-actin with 2 new 3.2-mm punches from the same NBS card.<br>If TREC values remained less than 25/μL and the β-actin levels were normal, confirming DNA template integrity, an abnormal report was issued and the primary care physician was contacted. At this point, the primary care physician could either request a confirmatory flow cytometry screening test to validate the diagnosis of T-cell lymphopenia, which is the option recommended by the NBS program, or obtain a new NBS card for a repeat TREC assay.

eTable 4. Classification of (case) definitions and outcomes after follow-up used in studies on NBS for SCID

Author (year)	Ref	Location	Classification	Definition
Thorsen et al. (2021)	(1)	Wisconsin	SCID (N=8)<br><br>Non-SCID & non syndromic T-cell lymphopenia (N=12)<br>Syndrome/chromosomal abnormality (N=14)<br>False positives (N=34)	SCID definitions were classified using published Primary Immune Deficiency Treatment Consortium (PIDTC) criteria (39)<br>Non-SCID T cell lymphopenia was defined as a CD3+ T cell number that was below the age-adjusted 10th percentile (TCL on confirmatory testing, but did not meet the criteria for SCID)<br>Syndromic non-SCID TCL<br>Normal for age T cell numbers and normal frequency of naïve CD4+ T cells.
Richards et al. (2020)	(2)	Australia and New Zealand	SCID<br>Typical SCID<br><br><br><br>Leaky/atypical SCID<br><br><br>Radiosensitive SCID<br><br><br><br>Omenn syndrome<br><br><br>Non SCID T cell lymphopenia<br><br>Non SCID T cell lymphopenia due to syndrome<br><br>Secondary T cell lymphopenia	 Absent or very low numbers of T cells (CD3+ T cells <300/uL) and no or very low T cell function (<10% lower range of normal), as measured by PHA or detectable TME<br>Reduced number of CD3+ T cells for age, less than 30% of lower limit of normal T cell function as measured by proliferation to PHA, reduced or absent naïve T cells and absence of TME<br>Genetic deficiencies in multiple genes required for DNA repair. T-B-NK+ immunophenotype due to the dependence on these genes for the generation of a functional T- and B cell antigen receptor during lymphocyte development<br>Caused by mutations in RAG1/2, but can be a result of many different gene mutations. Expansion of autologous dysregulated T cells and poor humoral immunity.<br><br>Typically have low T cells but naïve T cells are present (however may be low/absent), and ≥30% the lower limit of normal lymphocyte proliferation in response to PHA (note this will be method dependent e.g. whole blood versus separated PHA).<br>Typically have other physical features to suggest diagnose e.g. CHARGE<br>e.g. cardiac surgery, maternal medication
Gizewska et al. (2020)	(3)	Poland and Germany	SCID (N=1, CHH patient)<br>CID (N=1)<br>Agammaglobulinemia N=1	SCID defined as T-cells <300 cells/µL

			Nijmegen Breakage (N=1)<br>Transient B-cell lymphopenia immunosuppress. (N=1)<br>T/B-cell lymphopenia prematurity (N=1)<br>T- cell lymphopenia of unknown reason (N=1)<br>False positives (N=1)	False-positive results were defined when values for TRECs or KRECs in NBS were over the established cut-offs in absence of SCID or other PID in the confirmatory diagnosis
Blom et al. (2020)	(4)	The Netherlands	SCID (N=1)<br>T-cell impairment syndromes (N=5)<br><br>Secondary T-cell impairment (N=28)<br><br>Idiopathic T-cell lymphopenia (N=5)<br>False positives (N=8)	Absent naïve T-cells: ≤200 naïve/µl, not further specified<br>Congenital syndrome associated with T-cell impairment (low or abnormal T-cells: ≤1500 CD3+/µl and >200 naïve/µl)<br>T-cell lymphopenia attributed to other medical conditions without an intrinsic defect in the production of T-cells (low or abnormal T-cells: ≤1500 CD3+/µl and >200 naïve/µl)<br>T-cell lymphopenia with an unknown underlying cause (low or abnormal T-cells: ≤1500 CD3+/µl and > 200 naïve/µl)<br>Unknown underlying cause for the low TREC levels and normal flow cytometric results: >1500 CD3+/µl and >200 naïve/µl
Strand et al. (2020)	(5)	Norway	True positive SCID/CID (N=7)<br>Safety net<br>Possible SCID (also safety net)<br><br>Not SCID	Positive gene panel (TREC either <5/uL or 5-20 uL)<br>If TRECs are below 15/µL and NBS-NGS gene panel negative in an apparently healthy child with normal weight born to term, a second DBS sample is requested as a "safety net<br>Negative gene panel and TRECs < 5 uL or normal screening result
Argudo-Ramírez et al. (2019)	(6)	Catalonia (Spain)	SCID (N=1)<br><br><br>Non SCID lymphopenia (N=13)<br><br><br>Transient lymphopenia (N=4)<br><br>False positives (N=9)	CD3 T Cells/µL <300 PHA proliferation ==< 10% of normal<br>Supporting features: Detectable maternal T cells in peripheral blood; proven deleterious defect(s) in a known SCID gene.<br>Lymphopenia without SCID criteria, including prematurity (N=2), idiopathic lymphocytopenia, DiGeorge (N=5), Down syndrome (N=1), chylothorax (N=2)<br>Initially low TRECs and low lymphocyte count, with recovery in the following months<br>Initially normal lymphocyte count with normalization of TRECs between 3 and 6 months of life
Thomas et al. (2019)<br>Audrain et al. (2018)	(7, 8)	France	SCID (N=3)<br>Leaky SCID (N=3)<br><br><br>Variant SCID (N=0)	Persistent lymphopenia; <300 autologous CD3+ T-cells/µL.<br>300–1499 autologous CD3+ T-cells/µL; associated with a genetic defect in a known SCID gene. Omenn syndrome includes erythroderma, hepatosplenomegaly, eosinophilia, and oligoclonal T-cells.

			Secondary T-cell impairment (N=15)<br><br>Syndromes with T-cell impairment (N=7)<br><br>Idiopathic lymphocytopenia (N=27)<br><br>Preterm birth alone (N=7)<br>Normal flow cytometry (N=78) / visit with no flow cytometry (N=13) / deaths with no flow cytometry (N=12)	300–1499 autologous CD3+ T-cells/μL; functional T-cell impairment; no defect in known SCID genes.<br>Presence of congenital malformation or disease process that causes greater loss of T-cells, e.g., congenital cardiac defects, gastroschisis, intestinal lymphangiectasia, or hydro<br>Genetic syndrome that includes impairment within its spectrum of clinical findings, e.g. DiGeorge syndrome or Down's syndrome.<br>Distinction between transient (N=17) and moderate (N=19). Not further specified.<br>Preterm infants with no preexisting conditions who have low T-cell levels.
Amatuni et al. (2019)	(9)	California	SCID (N=50)<br>- Typical SCID<br><br>- Leaky and/or Omenn syndrome<br><br>Non-SCID TCL<br>- Syndrome associated with T-cell impairment (N=72)<br>- Secondary TCL (N=25)<br>- TCL and preterm birth alone (N=33)<br>- Idiopathic TCL (N=33)<br><br>False positive (not specified in category)<br>(N=349)	300 autologous T cells per μL, absent naïve T cells, and proliferative responses to the mitogen phytohemagglutinin that are <10% of control values.<br><1500 autologous T cells per μL unless there has been oligoclonal proliferation of memory phenotype T cells, as occurs in Omenn syndrome<br><br>Non-SCID TCL of <1500 T cells per μL<br>Syndrome associated with T-cell impairment or of a non-SCID primary immunodeficiency disorder<br>Not further specified<br><br>Idiopathic cases for which an underlying condition could not be determined, even after immunologic evaluation and, in many instances, sequencing of gene panels or whole exome<br>Not specified
Kobrynski et al. (2019)	(10)	USA	SCID<br>Non-SCID TCL<br>- Syndromes with T-cell impairment<br>- T-cell loss or destruction<br>- False positive results	Genetic congenital defects causing impaired T-cell development<br>T-cell losses associated with certain congenital heart defects, with gastrointestinal conditions, such as gastroschisis, intestinal

				lymphangiectasia, in utero exposure to immunosuppressants, or T-cell destruction, caused by neonatal leukemia.<br>The term false-positive result denotes an abnormal test result in the absence of disease.
Nourizadeh et al. (2018)	(11)	Iran	No classification/no clinical follow-up	No follow-up due to the lack of access to the infants and the possibility to obtain new samples and collect demographic, clinical and laboratory data. Samples with low TRECS are considered false positive results or they may in fact be immunodeficient patients
Al-Mousa et al. (2018)	(12)	Saudi Arabia	No classification/ no clinical follow-up	No clinical follow-up of screen positive cases was possible and to ensure the identification of all newborns with classical SCID and possibly other combined immunodeficiencies with low TREC
Routes, Verbsy et al. (2018)	(13)	USA Wisconsin	SCID (9%)<br><br>Leaky SCID<br><br><br>Congenital syndrome (34%)<br>Secondary (29%)<br>Unspecified (26%)<br><br>Idiopathic T cell lymphopenia (3%)<br>*Data from (29)*	T cell count < 300/mm and T cell proliferation assay (mitogen assay) less than 10% of control<br>Leaky SCID is T cell lymphopenia caused by hypomorphic mutations in SCID-causing genes associated with a T cell count > 300/mm and abnormal T cell function.<br>Congenital syndromes associated with T cell lymphopenia<br>Secondary causes of T cell lymphopenia are most commonly caused by egress of lymphocytes into the extravascular space<br>Idiopathic T cell lymphopenia, which is also known as variant SCID, refers to T cell lymphopenia with a T cell count greater than 300/mm without a known cause
Rechavi et al. (2017)	(14, 15)	Israel	SCID (N=5)<br>Leaky SCID (N=3)<br><br>Syndromic patients (N=9)<br>Prematurity (N=9)<br>Lymphopenia due to secondary cause (N=4)<br>False positives (N=11)<br><br><br>Unknown etiology (N=5)	Defined by us as less than 300/µl CD3+ T cells in peripheral blood<br>T lymphopenia but >300/µl CD3+ T cells<br><br>Congenital syndromes with variable degrees of T-cell impairment<br>Extreme prematurity with slow recovery of the immune system<br>Secondary T cell immunodeficiency<br><br>Newborns with consecutive positive screening results, whose clinical presentation was unremarkable and immunological workup was negative for lymphopenia of any etiology<br>Unclassifiable, confirmation tests were abnormal (thus excluding them as FP) By 1 year, all of these children had normal repeat workup. No medical intervention required
Son et al. (2017)	(16)	Korea	No classification	N=1 patient followed up at the clinic found to be healthy with no clinical issues

Kanegae et al. (2017)	(17)	Brazil	No classification	N= 1 patient died on the sixth day after birth as a result of a pleural effusion, N=1 patient loss to follow-up
Kanegae et al. (2016)	(18)	Brazil	No classification	N=1 patient loss to follow-up, N=3 patients normal flow cytometry
Tagliaferri et al. (2017)	(19)	Germany	No classification due to anonymized inclusion and no clinical follow-up	
Barbaro et al. (2017)	(20)	Sweden	PID (N=3)<br>Maternal immunosuppression (N=13)<br>Twin/triplet (N=11)<br>Premature (N=24)<br>Spontaneously normalized (N=29)<br>Declined resampling (N=4)	Considered to have a severe immunodeficiency disorder<br>Infants born to mothers receiving immunosuppressive therapy<br><br><37 weeks gestation<br>No apparent cause identified and the TREC/KREC levels in the children tested normalized with time
Zetterström et al. (2017)	(21)	Sweden	PID (N=5)<br>Premature (N=37)<br>Maternal immunosuppression (N=19)<br>Died prior to re-sampling (N=6)<br>Declined follow-up (N=1)	Confirmed immune deficiency<br><37 weeks gestation<br>Mothers had been receiving immunosuppressive therapy
De Felipe et al. (2017)<br>De Felipe et al. (2016)	(22, 23)	Spain	No exact classification, but one case fatal chromosomopathy, extreme premature newborns (N=2); neonates were born to mothers receiving azathioprine during pregnancy (N=2)	False-positive results were defined as values below the established cut-offs for TRECs or KRECs in absence of SCID or inherited agammaglobulinemia, respectively
Blom et al. (2017)	(24)	The Netherlands	No classification due to anonymized inclusion and no clinical follow-up	
Chien et al. (2017)	(25)	Taiwan	No. with T lymphopenia (N=136)<br>No. with SCID (N=7)<br>No. with variant SCID (N=8)<br>No. with 22q11.2 deletion (N=20)<br>No. of T cell loss (N=24)<br>No. of premature infants (N=59)<br>No. with other conditions (N=14)	Not specified, but cases of T-cell lymphopenia were attributed to T cell loss include sampling after operations for congenital heart diseases, volvulus, and congenital diaphragmatic hernia. Other conditions included maternal HIV, maternal systemic lupus erythematosus or other autoimmune disorders, Down syndrome, chromosome anomalies other than 22q11.2 deletion, leukemia etc.
Madkaikar et al. (2016)	(26)	India	Typical SCID<br><br>Leaky SCID or Omenn syndrome	<300 autologous T cells/µl of blood and <10 % of normal proliferation to mitogens [e.g., PHA]

			Variant SCID<br><br>Conditions with primary T cell lymphopenia<br><br>Secondary T cell lymphopenia<br><br><br>Premature infants presenting with T cell lymphopenia	Mutations in typical SCID genes that do not completely abolish gene function, 300 to 1500 autologous T cells/µl. Omenn syndrome: may have normal/elevated CD3 T cell counts but restricted TCR diversity (oligoclonality) of T cells.<br>Variant SCID with persistently low T-cells but no defect in a known SCID gene<br>CD3 T cells ≤ 1500 cells/uL) e.g. DiGeorge syndrome, CHARGE Jacobsen, Trisomy 21 etc.<br><br>Subset of infants with recognized congenital conditions, e.g. intestinal lymphangiectasia, hydrops, gastroschisis, a congenital heart defect, chylothorax, neonatal leukemia, prenatal administration of glucocorticoids or inflammatory conditions (e.g., sepsis).<br>Preterms with T cells ≤1500 cells/ul, resolves with age
Chien et al. (2015)	(27)	Taiwan	SCID (N=2)<br>SCID variants (N=2)<br>22q11.2 deletion microdeletion syndrome (N=5)<br>Other medical conditions (N=9)<br>Negative (N=6)	SCID according to CLSI 2011.<br>Idiopathic T-cell lymphopenia / T cell lymphopenia according to CLSI 2011<br>Chromosome 22q.11.2 microdeletion syndrome<br><br>Congenital heart disease (N=5), CMV (N=1), extreme low birth weight (N=3)<br>Not specified (normal flow cytometry)
Kwan et al. (2015)	(28)	Navajo Nation	Pilot: TCL (N=1) and no follow-up (N=1)<br>Population based: SCID-A (N=4)	SCID Artemis
Kwan et al. (2014)	(29)	USA	Typical SCID (N=42)<br><br><br>Leaky SCID (N=9)<br><br>Omenn syndrome (N=1)<br><br><br><br>Syndromes with low T-cell numbers/ T-cell impairment (N=136)<br>Secondary T-cell lymphopenia (N=117)	<300 (autologous CD3 T-cells/uL), proliferation to PHA <10% as adopted by PICTC and R4S Laboratory Performance Database, detectable maternal T cells in peripheral blood; proven deleterious defect(s) in a known SCID gene<br>300-1500, few naïve T cells, reduced (10%-50% of normal), no maternal T cells detectable; incomplete defect(s) in a known SCID gene<br>Oligoclonal T cells, reduced (10%-50% of normal), erythroderma, hepatosplenomegaly, eosinophilia, and elevated levels of serum IgE antibody<br><br>Recognized genetic syndrome that includes low T-cell numbers within its spectrum of clinical findings

			Preterm birth alone (N=29)<br><br>Idiopathic T-cell lymphopenia (variant SCID) (N=12)<br><br>Unspecified T-cell lymphopenia<br>False positives	Congenital malformation or disease process without an intrinsic defect in production of circulating T cells<br>Preterm birth and low birth weight, with low T-cell numbers early in life that normalize over time<br>Low T-cell numbers without recognized cause; 6 programs used 300-1500 autologous T cells/μL plus evidence of functional immune cell impairment, while other programs included infants with higher T-cell numbers<br>No further information available<br>Nonnormal TREC results that require a follow-up flow cytometry test, which when performed shows T cells above the program cutoff for T-cell lymphopenia
Audrain et al. (2014)	(30)	France	No classification due to anonymized inclusion and no clinical follow-up	
Adams et al. (2014)	(31)	UK	No classification due to anonymized inclusion and no clinical follow-up	
Vogel et al. (2014)	(32)	New York	Normal CBC and flow cytometry (N=381)<br>Clinical significant condition (N=97)<br>Pending further evaluation (N=14)<br>No longer referral due to addition of borderline category (N=14)<br>Expired, no diagnosis (N=16)<br>Lost to follow-up (N=8)<br>Parental refusal (N=1)	Classic SCID (N=9)<br>Leaky SCID (N=1)<br>Idiopathic T-cell lymphopenia of the newborn (N=19: newborns without SCID, a birth defect or another syndrome, who required ongoing monitoring or treatment for a deficiency of T-cells<br>Syndrome with T-cell impairment (N=11)<br>Secondary T-cell lymphopenia other than preterm (N=17) secondary T-cell lymphopenia as a complication of a major birth defect or surgical thymectomy<br>Other (N=13) Other laboratory abnormalities were identified in 13 infants. Absolute T-cell counts (CD3) were normal on flow cytometry; however, these infants exhibited other immune abnormalities
Kwan et al. (2013)	(33)	California	SCID<br>- Typical SCID (N=11)<br>- Leaky SCID/Omenn (N=3)<br>- Complete DiGeorge (N=1)<br>Variant SCID/CID (N=6)<br>Syndromes associated with TCL (n=12)<br>Secondary T lymphopenia to other condition (N=9)<br>Preterm (N=8)	TCL defined as <1,500 CD3 T cells/μL, or absence or marked reduction in CD4 naïve T cells (CD4/CD45RA), defined as <5% total CD3 T cells<br><br><br>Defined as TCL with functional T cell impairment for >3 months, without syndromic features or defects in a known SCID gene<br>Congenital syndromes with variable degrees of T cell impairment<br>TCL was also found secondary to congenital or postnatal major congenital heart disease (N=6), gastrointestinal malformations (N=3),

			False positive (N=111)	hydrops (N=2), multiple anomalies without a unifying diagnosis (N=2) and chylothorax (N=1)<br>False positives, samples that were “positive” or “incomplete” by TREC test but subsequently normal by flow cytometry
Borte et al. (2012)	(34)	Sweden	No classification due to anonymized inclusion and no clinical follow-up	
Verbsky et al. (2011)	(35)	Wisconsin	SCID/severe TCL (N=5)<br>Secondary causes for TCL (N=19)<br><br><br>Primary TCL including reversible TCL (N=9)<br>False positives/ Normal (N=38)	 Anatomic abnormalities of the lymphatics, chromosomal abnormalities, multiple congenital anomalies, or a presumed metabolic disorder<br>T-cell lymphopenia resolved (N=5), 22q11 deletion syndrome (N=4)<br><br>No TCL
Comeau et al. (2010)	(36)	Massachusetts	WNL flow cytometry results (N=5)<br>Flow cytometry or further repeat NBS results pending (N=1)<br>Flow cytometry not done, functional testing normal (N=1)<br>Monitored with serial NBS specimens and resolved with eventual normal NBS (N=14)<br>Documented thymectomy and previous normal SCID NBS result prior to thymectomy; multiple NBS specimens submitted after thymectomy and no further testing was recommended (N=2)<br>Expired prior to further testing (includes 3 infants in whom diagnosis of SCID or other PI had not been excluded) (N=8)<br>Lost to follow-up (infant moved out of country) (N=1)<br>Flow cytometry results indicating T cell lymphopenia (N=17)	Cases with T cell lymphopenia<br>4 DiGeorge syndrome<br>1 Jacobsen syndrome (11q deletion)<br><br>1 multiple congenital anomalies with unspecified T cell lymphopenia possibly due to failed chemical abortion with methotrexate for suspected ectopic pregnancy<br>2 thymectomies at cardiac surgery<br>9 normal lymphocyte function testing (diagnosis of SCID excluded) but continue to show idiopathic T cell lymphopenia and remain under the care of an immunologist.<br>1 pending, SCID unlikely though not yet excluded<br>1 not SCID, expired due to cardiac complications
Baker et al. (2010)	(37, 38)	Wisconsin	Normal flow cytometry (N=3)<br>Abnormal flow cytometry (N=8)<br>- DiGeorge syndrome (N=2)	Specimens that are normal on repeat testing (either filter paper or flow) are considered screening false positives.<br>Not further specified

Routes et al. (2009)			- Extravasation of T -cells outside vascular space (N=3)<br>-Idiopathic lymphocytopenia (N=3)	

eTable 5. Terminology and definitions of preterm infants used in studies on NBS for SCID

Author (year)	Ref	Location	Terminology used	Definition	Adjustment in screening algorithm?
Thorsen et al. (2021)	(1)	Wisconsin	Preterm	Not specified	Premature infants follow a distinct previously reported follow-up algorithm (35)
Richards et al. (2020)	(2)	Australia and New Zealand	NR	NR	NR
Gizewska et al. (2020)	(3)	Poland and Germany	Preterm newborns	≥38 weeks—born at term; ≥32–37 weeks—moderate preterm, ≥28–32 weeks—very preterm; <28 weeks—extremely preterm	In the case of extremely and very preterm newborn (born <32 HBD) the second screening cards were taken when the child reached 32–34 weeks of gestational age
Blom et al. (2020)	(4)	The Netherlands	Preterm infant	Gestational age < 37 weeks and birth weight ≤ 2500 gram	If TREC ≤ cut-off, second DBS from the corrected gestational age of 37 weeks
Strand et al. (2020)	(5)	Norway	Premature	Gestational age <35 weeks	Different cut-ff value for preterms: second tier NGS for TREC 5-15 uL instead of 5-20 uL
Argudo-Ramírez et al. (2019)	(6)	Catalonia (Spain)	Preterm newborns	Gestational age <37 weeks	Different cut-off value for preterms: after duplicate analysis TREC 6-20: second DBS after 37 weeks instead of full terms 11-20 second DBS immediately.
Thomas et al. (2019)<br>Audrain et al. (2018)	(7, 8)	France	Preterm	Gestational age <37 weeks	New DBS samples if preterms tested presumptive positive with a TREC value between 5 copies/μL and the second cutoff. Different cut-off used for preterms.
Amatuni et al. (2019)	(9)	California	Preterm	Not specified	No specific for preterms, but for infants in NICU: Second DBS test in 2 wk or at discharge from nursery; after 2 incomplete test results, liquid blood for lymphocyte subsets
Kobrynski et al. (2019)	(10)	USA	Premature infants	<35 weeks	For premature infants (<2500 g), most states collect new DBS samples and repeat TREC testing at intervals, until term gestation or the TREC level normalizes. Undetectable (less than first percentile) TREC level or whose TREC test

					result fails to normalize by 37 weeks gestational age need flow cytometry
Nourizadeh et al. (2018)	(11)	Iran	NR	NR	NR
Al-Mousa et al. (2018)	(12)	Saudi Arabia	Preterm newborns	<37 weeks gestation	Not in this pilot study
Routes, Verbsy et al. (2018)	(13)	USA Wisconsin	Preterm	Not specified	Different policies in different states. Wisconsin: TREC assay is reflexively performed on premature infants until they have reached an adjusted gestational age over 36 weeks
Rechavi et al. (2017)	(14, 15)	Israel	Preterms	<37 weeks gestation	In early preterms, a lower cut-off is used to determine the need for retesting. As such, so long as a preterm infant is hospitalized, a new, repeat Guthrie card is tested every two weeks
Son et al. (2017)	(16)	Korea	NR	NR	NR
Kanegae et al. (2017)	(17)	Brazil	Preterm	26-36 weeks	Preterm infants with altered TRECs values at birth should undergo a second newborn screening for T-cell lymphopenia at an adjusted gestational age of 37 weeks
Kanegae et al. (2016)	(18)	Brazil	Preterm	30-36 weeks	Not in this pilot study
Tagliaferri et al. (2017)	(19)	Germany	NR	NR	NR
Barbaro et al. (2017)	(20)	Sweden	Premature	Prior to 37 weeks gestation	In premature inpatient children, contact with neonatologist. In the beginning of the study a follow-up DBS sample was taken immediately at recall, and then again after the first year of the study, at 37 week´s gestational age, or when the child was discharged from the ward if there was no suspicion of an immune deficiency

Zetterström et al. (2017)	(21)	Sweden	Premature	Gestational age <37 weeks	See Barbaro et al. (2017) (20)
De Felipe et al. (2017)<br>De Felipe et al. (2016)	(22, 23)	Spain	Premature	GA < 37 weeks	Not in this pilot study
Blom et al. (2017)	(24)	The Netherlands	Preterm newborns	Birthweight ≤ 2500 gram and gestational age ≤ 36.0 weeks	Suggestions for adjustment screening algorithm are mentioned in text, but not applied
Chien et al. (2017)	(25)	Taiwan	Preterm infants	<37 weeks (not clearly specified)	The same cut-offs are used for term and preterm infants, but the preterm infants are re-tested at 37 weeks of gestational age
Madkaikar et al. (2016)	(26)	India	Premature infants	Not specified	NA
Chien et al. (2015)	(27)	Taiwan	Preterm infants	Not specified	Not in this pilot study
Kwan et al. (2015)	(28)	Navajo Nation	Preterm birth	<37 weeks	Not in pilot study, but in population based screening inconclusive, collect repeat DBS in 4 weeks or at 37 weeks gestation
Kwan et al. (2014)	(29)	USA	Preterm	Not specified	Programs did not report preterm infants with low T cells in a uniform manner, partly due to automatically repeated TREC testing of preterm infants in neonatal intensive care units in some screening program
Audrain et al. (2014)	(30)	France	NR	NR	NR
Adams et al. (2014)	(31)	UK	Preterm babies	<36 weeks	Not in this pilot study
Vogel et al. (2014)	(32)	New York	Premature infants	<37 weeks gestation	≤200 TRECs who were born prematurely (a repeat specimen was requested at an age equivalent to at least 37 weeks gestation. Infants with undetectable TRECs were referred for a

					diagnostic evaluation, regardless of gestational age
Kwan et al. (2013)	(33)	California	Preterm birth/infants	Not specified	No specific for preterms, but for infants in NICU: Second DBS test in 2 wk or at discharge from nursery; after 2 incomplete test results, liquid blood for lymphocyte subsets
Borte et al. (2012)	(34)	Sweden	Prematurity	Not specified	Not in this pilot study
Verbsky et al. (2011)	(35)	Wisconsin	Preterm	<37 weeks	For pre-term infants (AGA <37 weeks) with an abnormal or inconclusive TREC assay, the screening test was repeated until either normal or until the infant reached 37 weeks AGA at which time the infant was reclassified as an abnormal
Comeau et al. (2010)	(36)	Massachusetts	Pre-term infants	Not specified	In some cases: monitored with serial NBS specimens, and lymphocyte phenotypic and functional testing be undertaken as soon as possible
Baker et al. (2010)<br><br>Routes et al. (2009)	(37, 38)	Wisconsin	Preterm	<37 weeks	All preterm infants (<37 weeks' gestation) with abnormal or inconclusive TREC assays have their TREC levels monitored until the infant reaches the equivalent of 37 weeks' gestation; then full-term criteria are applied

*NR not reported, NA not applicable

Online-Only References

1. Thorsen J, Kolbert K, Joshi A, Baker M, Seroogy CM. Newborn Screening for Severe Combined Immunodeficiency: 10-Year Experience at a Single Referral Center (2009-2018). J Clin Immunol. 2021.

2. Richards S, Gennery AR, Davies EG, Wong M, Shaw PJ, Peake J, et al. Diagnosis and management of severe combined immunodeficiency in Australia and New Zealand. Journal of Paediatrics and Child Health. 2020;56(10):1508-13.

3. Giżewska M, Durda K, Winter T, Ostrowska I, Ołtarzewski M, Klein J, et al. Newborn Screening for SCID and Other Severe Primary Immunodeficiency in the Polish-German Transborder Area: Experience From the First 14 Months of Collaboration. Front Immunol. 2020;11:1948.

4. Blom M, Bredius RGM, Jansen ME, Weijman G, Kemper EA, Vermont CL, et al. Parents' Perspectives and Societal Acceptance of Implementation of Newborn Screening for SCID in the Netherlands. J Clin Immunol. 2021;41(1):99-108.

5. Strand J, Gul KA, Erichsen HC, Lundman E, Berge MC, Trømborg AK, et al. Second-Tier Next Generation Sequencing Integrated in Nationwide Newborn Screening Provides Rapid Molecular Diagnostics of Severe Combined Immunodeficiency. Front Immunol. 2020;11:1417.

6. Argudo-Ramírez A, Martín-Nalda A, Marín-Soria JL, López-Galera RM, Pajares-García S, González de Aledo-Castillo JM, et al. First Universal Newborn Screening Program for Severe Combined Immunodeficiency in Europe. Two-Years' Experience in Catalonia (Spain). Front Immunol. 2019;10:2406.

7. Thomas C, Durand-Zaleski I, Frenkiel J, Mirallié S, Léger A, Cheillan D, et al. Clinical and economic aspects of newborn screening for severe combined immunodeficiency: DEPISTREC study results. Clinical Immunology. 2019;202:33-9.

8. Audrain MAP, Léger AJC, Hémont CAF, Mirallié SM, Cheillan D, Rimbert MGM, et al. Newborn Screening for Severe Combined Immunodeficiency: Analytic and Clinical Performance of the T Cell Receptor Excision Circle Assay in France (DEPISTREC Study). Journal of Clinical Immunology. 2018;38(7):778-86.

9. Amatuni GS, Currier RJ, Church JA, Bishop T, Grimbacher E, Nguyen AA, et al. Newborn Screening for Severe Combined Immunodeficiency and T-cell Lymphopenia in California, 2010-2017. Pediatrics. 2019;143(2).

10. Kobrynski LJ. Identification of non–severe combined immune deficiency T-cell lymphopenia at newborn screening for severe combined immune deficiency. Annals of Allergy, Asthma & Immunology. 2019;123(5):424-7.

11. Nourizadeh M, Shakerian L, Borte S, Fazlollahi M, Badalzadeh M, Houshmand M, et al. Newborn screening using TREC/KREC assay for severe T and B cell lymphopenia in Iran. Scand J Immunol. 2018:e12699.

12. Al-Mousa H, Al-Dakheel G, Jabr A, Elbadaoui F, Abouelhoda M, Baig M, et al. High Incidence of Severe Combined Immunodeficiency Disease in Saudi Arabia Detected Through Combined T Cell Receptor Excision Circle and Next Generation Sequencing of Newborn Dried Blood Spots. Front Immunol. 2018;9:782.

13. Routes J, Verbsky J. Newborn Screening for Severe Combined Immunodeficiency. Current Allergy and Asthma Reports. 2018;18(6):34.
14. Rechavi E, Lev A, Simon AJ, Stauber T, Daas S, Saraf-Levy T, et al. First Year of Israeli Newborn Screening for Severe Combined Immunodeficiency-Clinical Achievements and Insights. Front Immunol. 2017;8:1448.
15. Rechavi E, Lev A, Saraf-Levy T, Etzioni A, Almashanu S, Somech R. Newborn Screening for Severe Combined Immunodeficiency in Israel. International Journal of Neonatal Screening. 2017;3(2):13.
16. Son S, Kang J-M, Kim JM, Sung S, Kim Y-S, Lee H, et al. The First Newborn Screening Study of T-Cell Receptor Excision Circle and κ-Deleting Recombination Excision Circle for Severe Combined Immunodeficiency in Korea: A Pilot Study. Pediatr Infect Vaccine. 2017;24(3):134-40.
17. Kanegae MPP, Barreiros LA, Sousa JL, Brito MAS, Oliveira EBJ, Soares LP, et al. NEWBORN SCREENING FOR SEVERE COMBINED IMMUNODEFICIENCIES USING TRECS AND KRECS: SECOND PILOT STUDY IN BRAZIL. Rev Paul Pediatr. 2017;35(1):25-32.
18. Kanegae MPP, Barreiros LA, Mazzucchelli JTL, Hadachi SM, de Figueiredo Ferreira Guilhoto LM, Acquesta AL, et al. Neonatal screening for severe combined immunodeficiency in Brazil. Jornal de Pediatria. 2016;92(4):374-80.
19. Tagliaferri L, Kunz JB, Happich M, Esposito S, Bruckner T, Hübschmann D, et al. Newborn screening for severe combined immunodeficiency using a novel and simplified method to measure T-cell excision circles (TREC). Clinical Immunology. 2017;175:51-5.
20. Barbaro M, Ohlsson A, Borte S, Jonsson S, Zetterström RH, King J, et al. Newborn Screening for Severe Primary Immunodeficiency Diseases in Sweden-a 2-Year Pilot TREC and KREC Screening Study. J Clin Immunol. 2017;37(1):51-60.
21. Zetterström RH, Barbaro M, Ohlsson A, Borte S, Jonsson S, Winiarski J, et al. Newborn Screening for Primary Immune Deficiencies with a TREC/KREC/ACTB Triplex Assay—A Three-Year Pilot Study in Sweden. International Journal of Neonatal Screening. 2017;3(2):11.
22. De Felipe B, Olbrich P, Goycochea-Valdivia W, Delgado-Pecellin C, Sanchez-Moreno P, Sánchez B, et al. Newborn Screening for Primary T- and B-Cell Immune Deficiencies—A Prospective Study in Andalucía. International Journal of Neonatal Screening. 2017;3(4):27.
23. de Felipe B, Olbrich P, Lucenas JM, Delgado-Pecellin C, Pavon-Delgado A, Marquez J, et al. Prospective neonatal screening for severe T- and B-lymphocyte deficiencies in Seville. Pediatr Allergy Immunol. 2016;27(1):70-7.
24. Blom M, Pico-Knijnenburg I, Sijne-van Veen M, Boelen A, Bredius RGM, van der Burg M, et al. An evaluation of the TREC assay with regard to the integration of SCID screening into the Dutch newborn screening program. Clin Immunol. 2017;180:106-10.
25. Chien Y-H, Yu H-H, Lee N-C, Ho H-C, Kao S-M, Lu M-Y, et al. Newborn Screening for Severe Combined Immunodeficiency in Taiwan. International Journal of Neonatal Screening. 2017;3(3):16.
26. Madkaikar M, Aluri J, Gupta S. Guidelines for Screening, Early Diagnosis and Management of Severe Combined Immunodeficiency (SCID) in India. The Indian Journal of Pediatrics. 2016;83(5):455-62.

27. Chien YH, Chiang SC, Chang KL, Yu HH, Lee WI, Tsai LP, et al. Incidence of severe combined immunodeficiency through newborn screening in a Chinese population. J Formos Med Assoc. 2015;114(1):12-6.
28. Kwan A, Hu D, Song M, Gomes H, Brown DR, Bourque T, et al. Successful newborn screening for SCID in the Navajo Nation. Clin Immunol. 2015;158(1):29-34.
29. Kwan A, Abraham RS, Currier R, Brower A, Andruszewski K, Abbott JK, et al. Newborn screening for severe combined immunodeficiency in 11 screening programs in the United States. Jama. 2014;312(7):729-38.
30. Audrain M, Thomas C, Mirallie S, Bourgeois N, Sebille V, Rabetrano H, et al. Evaluation of the T-cell receptor excision circle assay performances for severe combined immunodeficiency neonatal screening on Guthrie cards in a French single centre study. Clin Immunol. 2014;150(2):137-9.
31. Adams SP, Rashid S, Premachandra T, Harvey K, Ifederu A, Wilson MC, et al. Screening of neonatal UK dried blood spots using a duplex TREC screening assay. J Clin Immunol. 2014;34(3):323-30.
32. Vogel BH, Bonagura V, Weinberg GA, Ballow M, Isabelle J, DiAntonio L, et al. Newborn screening for SCID in New York State: experience from the first two years. J Clin Immunol. 2014;34(3):289-303.
33. Kwan A, Church JA, Cowan MJ, Agarwal R, Kapoor N, Kohn DB, et al. Newborn screening for severe combined immunodeficiency and T-cell lymphopenia in California: results of the first 2 years. J Allergy Clin Immunol. 2013;132(1):140-50.
34. Borte S, von Döbeln U, Fasth A, Wang N, Janzi M, Winiarski J, et al. Neonatal screening for severe primary immunodeficiency diseases using high-throughput triplex real-time PCR. Blood. 2012;119(11):2552-5.
35. Verbsky JW, Baker MW, Grossman WJ, Hintermeyer M, Dasu T, Bonacci B, et al. Newborn screening for severe combined immunodeficiency; the Wisconsin experience (2008-2011). J Clin Immunol. 2012;32(1):82-8.
36. Comeau AM, Hale JE, Pai S-Y, Bonilla FA, Notarangelo LD, Pasternack MS, et al. Guidelines for implementation of population-based newborn screening for severe combined immunodeficiency. Journal of Inherited Metabolic Disease. 2010;33(S2):273-81.
37. Baker MW, Laessig RH, Katcher ML, Routes JM, Grossman WJ, Verbsky J, et al. Implementing routine testing for severe combined immunodeficiency within Wisconsin's newborn screening program. Public Health Rep. 2010;125 Suppl 2(Suppl 2):88-95.
38. Routes JM, Grossman WJ, Verbsky J, Laessig RH, Hoffman GL, Brokopp CD, et al. Statewide newborn screening for severe T-cell lymphopenia. Jama. 2009;302(22):2465-70.
39. Shearer WT, Dunn E, Notarangelo LD, Dvorak CC, Puck JM, Logan BR, et al. Establishing diagnostic criteria for severe combined immunodeficiency disease (SCID), leaky SCID, and Omenn syndrome: The Primary Immune Deficiency Treatment Consortium experience. Journal of Allergy and Clinical Immunology. 2014;133(4):1092-8.
